# Effective targeting of intact and proteolysed CDCP1 for imaging and treatment of pancreatic ductal adenocarcinoma

**DOI:** 10.7150/thno.43589

**Published:** 2020-03-04

**Authors:** Thomas Kryza, Tashbib Khan, Simon Puttick, Chao Li, Kamil A Sokolowski, Brian WC Tse, Tahleesa Cuda, Nicholas Lyons, Madeline Gough, Julia Yin, Ashleigh Parkin, Elena I Deryugina, James P. Quigley, Ruby H. P. Law, James C. Whisstock, Andrew D. Riddell, Andrew P. Barbour, David K. Wyld, Paul A. Thomas, Stephen Rose, Cameron E. Snell, Marina Pajic, Yaowu He, John D. Hooper

**Affiliations:** 1 Mater Research Institute - The University of Queensland, Translational Research Institute, Woolloongabba, QLD, Australia; 2 Commonwealth Scientific and Industrial Research Organisation, Herston, QLD, Australia; 3 China Medical University, Shenyang, China; 4 Preclinical Imaging Facility, Translational Research Institute, Woolloongabba, QLD, Australia; 5 Mater Health Services, South Brisbane, QLD, Australia; 6 The Kinghorn Cancer Centre, The Garvan Institute of Medical Research, Sydney, NSW, Australia; 7 St Vincent's Clinical School, Faculty of Medicine, University of NSW Sydney, NSW, Australia; 8 The Scripps Research Institute, La Jolla, CA, USA; 9 Biomedicine Discovery Institute and Department of Biochemistry and Molecular Biology, Monash University, Melbourne, VIC, Australia; 10 Faculty of Medicine, The University of Queensland, St. Lucia, QLD, Australia; 11 Redcliffe Hospital, Metro North Hospital and Health Service, Brisbane, QLD, Australia; 12 Upper Gastrointestinal/Soft Tissue Unit, Princess Alexandra Hospital, Woolloongabba, QLD, Australia; 13 Diamantina Institute, The University of Queensland, Woolloongabba, QLD, Australia; 14 Royal Brisbane and Women's Hospital, Herston, QLD, Australia; 15 Herston Imaging Research Facility, Herston, QLD, Australia

**Keywords:** pancreatic cancer, theranostics, monoclonal-antibody, PET-CT, CDCP1

## Abstract

**Background**: CUB domain-containing protein 1 (CDCP1) is a cell surface receptor regulating key signalling pathways in malignant cells. CDCP1 has been proposed as a molecular target to abrogate oncogenic signalling pathways and specifically deliver anti-cancer agents to tumors. However, the development of CDCP1-targeting agents has been questioned by its frequent proteolytic processing which was thought to result in shedding of the CDCP1 extracellular domain limiting its targetability. In this study, we investigated the relevance of targeting CDCP1 in the context of pancreatic ductal adenocarcinoma (PDAC) and assess the impact of CDCP1 proteolysis on the effectiveness of CDCP1 targeting agents.

**Methods**: The involvement of CDCP1 in PDAC progression was assessed by association analysis in several PDAC cohorts and the proteolytic processing of CDCP1 was evaluated in PDAC cell lines and patient-derived cells. The consequences of CDCP1 proteolysis on its targetability in PDAC cells was assessed using immunoprecipitation, immunostaining and biochemical assays. The involvement of CDCP1 in PDAC progression was examined by loss-of-function *in vitro* and *in vivo* experiments employing PDAC cells expressing intact or cleaved CDCP1. Finally, we generated antibody-based imaging and therapeutic agents targeting CDCP1 to demonstrate the feasibility of targeting this receptor for detection and treatment of PDAC tumors.

**Results**: High CDCP1 expression in PDAC is significantly associated with poorer patient survival. In PDAC cells proteolysis of CDCP1 does not always result in the shedding of CDCP1-extracellular domain which can interact with membrane-bound CDCP1 allowing signal transduction between the different CDCP1-fragments. Targeting CDCP1 impairs PDAC cell functions and PDAC tumor growth independently of CDCP1 cleavage status. A CDCP1-targeting antibody is highly effective at delivering imaging radionuclides and cytotoxins to PDAC cells allowing specific detection of tumors by PET/CT imaging and superior anti-tumor effects compared to gemcitabine in *in vivo* models.

**Conclusion**: Independent of its cleavage status, CDCP1 exerts oncogenic functions in PDAC and has significant potential to be targeted for improved radiological staging and treatment of this cancer. Its elevated expression by most PDAC tumors and lack of expression by normal pancreas and other major organs, suggest that targeting CDCP1 could benefit a significant proportion of PDAC patients. These data support the further development of CDCP1-targeting agents as personalizable tools for effective imaging and treatment of PDAC.

## Introduction

Pancreatic-ductal adenocarcinoma (PDAC), the most common form of pancreas cancer, is predicted by 2030 to become the second leading cause of cancer-related death in developed countries 1,2. Lack of clinical symptoms leading to diagnosis at late stage, difficulties in accurate staging, and limited treatment options contribute to the high mortality rate 1. While surgery significantly extends survival, marginal improvements are achieved with chemo- and radio-therapy, with overall five-year survival across the total patient cohort abysmal at less than 10% 2.

CUB Domain-Containing Protein 1 (CDCP1) is a single span transmembrane receptor 3 that relays cancer promoting signals via other receptors such as EGFR 4, HER2 5 and β1 integrin 6,7, as well as mediators of metabolism such as acyl CoA-synthetase 8 and key intracellular signal transducers including Src 9-12, PKCδ 13, Akt 6,13 and FAK 6,14. The activity of CDCP1 is regulated by limited proteolysis of its extracellular domain (ECD), converting the full-length (**FL**) 135 kDa glycoprotein to a 70 kDa membrane spanning carboxyl-terminal fragment (**CTF**) and an amino-terminal fragment (**ATF**) of 65 kDa that is shed from the surface of several prostate cancer cell lines 15,16 and detected in serum of colorectal cancer patients 17. CDCP1 proteolysis can modulate its interactions with molecular partners and the metastatic potential of cancer cells 6,8,15,16. Elevated expression of CDCP1 is associated with poor overall survival of patients with cancer of the breast 18-20, colorectum 21, kidney 22, lung 23, ovary 13,24 and pancreas 25 while a potential protective role has been recently identified in prostate cancer 7. Targeting CDCP1 using function blocking monoclonal antibodies inhibits vascular metastasis of prostate cancer 26, intraperitoneal progression of ovarian cancer 13,27 and subcutaneous growth of lung and breast cancer 28 in mouse xenograft models. In addition, the role of CDCP1 in stimulating lipid oxidation to promote triple-negative breast cancer metastasis in mouse models, can be markedly inhibited by stable over-expression of the CDCP1-ATF 29.

Limited experimental evidence suggests that CDCP1 is also important in PDAC. Immunohistochemical analysis of a single patient cohort revealed that elevated CDCP1 expression correlated with shorter overall survival although the result only demonstrated marginal significance possibly due to a lack of histological sub-classification of the patient cohort 25. Transient silencing of CDCP1 demonstrated that it regulates pancreatic cancer cell line migration, invasion, and extracellular matrix degradation 25. Upregulation of CDCP1 expression in response to transformation by *RAS* 30, an oncogene somatically mutated in more than 90% of PDAC tumors 31, is also suggestive of a functional role for CDCP1 in this cancer. The potential to target CDCP1 to induce killing of PDAC cells was demonstrated by a study which employed an antibody antigen binding fragment (F_AB_) that targeted this receptor. An immunoglobulin (IgG) incorporating this F_AB_ was employed as a bait to target an antibody-drug conjugate and activated T-cells to induce death of a PDAC cell line* in vitro*. Also, the F_AB_ conjugated with the positron-emitting radionuclide zirconium-89 (^89^Zr) was able to detect a subcutaneous PDAC cell line xenograft in mice by positron emission tomography-computed tomography (PET-CT) imaging although contrast was poor 30.

To define the potential of targeting CDCP1 in PDAC, in this study we demonstrate the association between CDCP1 expression level and patient survival in multiple PDAC patient cohorts. Secondly, we have examined the impact of proteolysis of its ECD on its pro-tumorigenic functions and targetability using *in vitro* and *in vivo* assays. Our data reveal for the first time stable interactions between CDCP1 proteolytic fragments and the possibility of signal transduction between CDCP1-ATF and CDCP1-FL/CTF. Importantly, our results indicate that proteolysis of the CDCP1 ECD does not alter the oncogenic functions of this receptor in PDAC or its ability to be an effective target for antibody-mediated abrogation of oncogenic signalling or delivery of imaging radionuclides and cytotoxins to PDAC tumors *in vivo*. Together our results indicate that CDCP1 is functionally important in PDAC and has clinical potential as a prognostic marker and target for delivery of agents for detection and treatment of this malignancy.

## Materials and Methods

Additional information is available in the Supplementary Materials and Methods file.

### Analysis of CDCP1 mRNA expression in PDAC tumors

Assessment of CDCP1 expression in PDAC was performed by analysis of mRNA datasets from The Cancer Genome Atlas (TCGA) and the International Cancer Genome Consortium (ICGC) that contain expression and overall survival data for 170 and 267 patients, respectively. These datasets are designated Pancreatic Adenocarcinoma - The Cancer Genome Atlas - Genomic Data Commons (PAAD-TCGA-GDC; abbreviated TCGA) and International Cancer Genome Consortium - Pancreatic Cancer - Australia (ICGC-PACA-AU; abbreviated ICGC). Data from the TCGA cohort were downloaded using the UCSC Xena browser 32 and for the ICGC cohort from the ICGC data portal 33. Clinical characteristics of patients are available in Table S1. CDCP1 mRNA expression was segregated into quartiles and different cut-off points (indicated in figure legends) were used to generate Kaplan-Meier curves with a Mantel-Cox test used to determine significance. The larger ICGC cohort was also used to test the association between clinical parameters (age, gender, tumor size, tumor stage, vasculature invasion, lymph node positivity) and CDCP1 expression level using ANOVA (Tumor size/stage) or t-test.

### Immunohistochemical analysis of CDCP1 expression in PDAC tumors

Tissue-microarrays containing specimens from 222 PDAC cases from the Australian ICGC PDAC cohort have been described previously 34,35. Clinical characteristics of patients are shown in Table S1. Patients had primary operable, untreated PDAC and underwent a pancreatectomy with tumor/normal specimens analysed by whole genome sequencing as part of the ICGC. Immunohistochemistry was performed as described previously using validated anti-CDCP1 antibody 4115 3,27. Staining was assessed by an anatomical pathologist (CES) blinded to clinical data. Staining was assessed using a semi quantitative scoring system of both the intensity (graded as 0, no staining; 1, weak; 2, moderate; or 3 strong) and percentage of positive cells (in 10% increments). The score assigned to each patient represented the average percentage of CDCP1 positive cells from two cores per patient. For generation of Kaplan-Meier survival curves, patient scores were dichotomised into those below or above the median score of the entire cohort which were then assigned to “low” and “high” CDCP1 expression groups, respectively. Additional analyses were performed using patient scores segregated into quartiles.

### Cell lines and patient-derived cells

CAPAN-1 and PANC-1 PDAC cell lines and HeLa cells were from ATCC (Manassas, VA) and cultured according to supplier protocols. Normal human pancreatic stellate cells (hPSC) were from ScienCell Research Laboratories (Carlsbad, CA). The APGI PDAC patient derived cells TKCC02 TKCC05, TKCC07, TKCC09, TKCC10, TKCC15, TKCC22, TKCC23 and TKCC27 were described previously 34,36. To avoid artificial cleavage of CDCP1, cell passages were performed non-enzymatically with Versene (0.48 mM EDTA in PBS, pH 7.4). Cells were cultured at 37°C in a humidified 5% CO_2_ atmosphere. TKCC02, TKCC10, TKCC15, TKCC22, TKCC23 and TKCC27 cells were cultured in reduced oxygen (5%). TKCC05, TKCC07, TKCC09, CAPAN-1 and PANC-1 cells were cultured in 20-21% oxygen. Using a previously described protocol, cells were stably transduced with a luciferase expression construct, one of two lentiviral CDCP1 silencing constructs (shCDCP1 #1, shCDCP1 #2) or a scramble control construct (shControl) 37. TKCC05 cells and hPSCs were stably transduced with a lentiviral vector (pLEX_307, Addgene #41392) encoding Monomeric Kusabira-Orange 2 (mKO2) and green fluorescent protein (GFP), respectively, as previously described 38.

### *In vivo* models

Mouse experiments were approved by the University of Queensland Animal Ethics Committee. PDAC cells were injected subcutaneously into the flanks (2.5×10^6^ in PBS) or into the mid-body of the pancreas (1×10^6^ in 1:1 PBS/Matrigel) of NOD.Cg-Prkdc^scid^ Il2rg^tm1Wjl^/SzJ (**NSG**) mice (6-8 weeks; Jackson Laboratory, Bar Harbor, ME). For assays assessing the impact of antibody 10D7 on subcutaneous xenograft growth, two weeks after PDAC cell inoculations, mice (n=6/group) were randomized and treated i.v. every four days with PBS, 10D7 (5 mg/kg) or IgG (5 mg/kg) until the end of the assay. For assays assessing whether 10D7 improves the efficacy of gemcitabine chemotherapy, four weeks after subcutaneous PDAC cell inoculations, mice were randomized and treated i.v. every four days with PBS (n=12), 10D7 (n=12, 5 mg/kg) or IgG (n=12, 5 mg/kg). Half of the mice in each of the three groups also received gemcitabine i.p. treatments (125 mg/kg/ week). At the end of the assay tumors were harvested, weighed and processed for assessment of histology and CDCP1 expression by immunohistochemistry or western blot analysis. For assays assessing the effect of MMAE-conjugated antibodies on subcutaneous xenograft growth and mouse survival, four weeks after PDAC cell inoculations, mice (8/group) were randomized and treated i.v. every two weeks with PBS, 10D7 (5 mg/kg), IgG (5 mg/kg), 10D7-MMAE (5 mg/kg) or IgG-MMAE (5 mg/kg), or weekly with i.p. administration of gemcitabine (125 mg/kg). Tumor burden was monitored by calliper measurement and tumor volume calculated as previously described 39. Tumor burden and weight results are presented as mean +/- SEM and statistical analysis was performed on the last data point using a Wilcoxon-Mann- Whitney test between groups.

### PET-CT imaging

PET-CT imaging was performed on NSG mice carrying subcutaneous or intra-pancreatic PDAC cell xenografts. Two weeks after subcutaneous PDAC cell inoculations and four weeks after intra-pancreas injections, mice received equivalent doses of either 10D7-^89^Zr or control IgG1κ-^89^Zr via the lateral tail vein (~1.5 MBq). PET-CT imaging was performed on isoflurane anaesthetised mice after 24, 48, 72 and 144 h using an Inveon PET/CT unit (Siemens, Munich, Germany). PET acquisition (30 minutes; static emission) was performed, and images were reconstructed using an ordered-subset expectation maximization (OSEM2D) algorithm, with CT attenuation correction. The CT scan parameters were 80 kV, 500 µA, 230 ms exposure time, 360^o^ rotation with 180 rotation steps, binning factor of 4, low magnification position, producing an effective pixel size of 106 µm, with CT images reconstructed using the Feldkamp algorithm. All PET and CT images were reconstructed using Inveon Acquisition Workplace software (Siemens). PET activity per voxel was converted to bq/cc using a conversion factor obtained by scanning a cylindrical phantom filled with a known activity of ^89^Zr to account for PET scanner efficiency. Activity concentrations within tissue ROIs were expressed as percentage of the decay-corrected injected activity per cubic cm of tissue (%ID/cc; SUV) using Inveon Research Workplace software (Siemens). *Ex vivo* bio-distribution was assessed after the final imaging time point. Harvested tumor and organs, cleaned of blood, were weighed and radioactivity quantified using a Wizard 2480 gamma counter (Perkin Elmer) and presented as %ID/g of tumor or tissue (after decay and detector efficiency corrections).

### Statistical Analysis

Statistical tests were performed using IBM SPSS Statistics 23 software (IBM Australia Ltd, St Leonards, Australia) and R (version 3.5.1; The R Foundation, www.r-project.org). Except where noted, the Mann- Whitney test was used in analysis comparing two groups while the Kruskal-Wallis test was used for comparisons involving more than two groups. A value of p ≤ 0.05 was considered significant. Significance values are represented in graphs as *p < 0.05, **p < 0.01, ***p < 0.001 and ****p < 0.0001.

## Results

### Elevated expression of CDCP1 is associated with poor PDAC patient outcome

**Figure 1:**
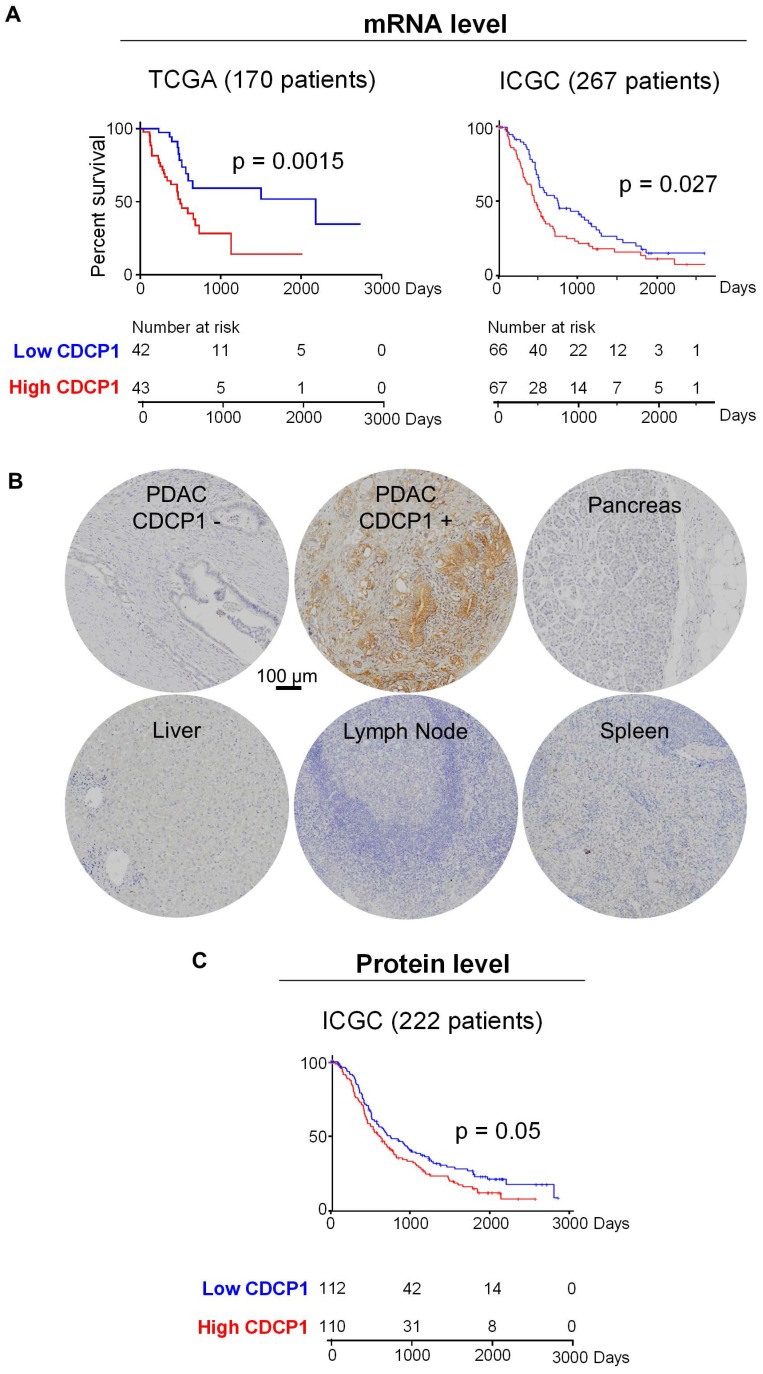
CDCP1 expression in PDAC tumors. **A,** Kaplan-Meier analysis showing association between CDCP1 mRNA expression levels and PDAC patient survival in TCGA (n=170) and ICGC (n=267) datasets. Patients in each dataset with CDCP1 mRNA expression levels in the first and fourth quartile were segregated into low and high CDCP1 expressing groups, respectively. **B,** Example images of CDCP1 immunohistochemical staining, using antibody 4115, of PDAC patient tumors and normal tissues. **C,** Kaplan-Meier analysis showing association between CDCP1 protein expression levels and PDAC patient survival in the ICGC-PACA-AU (n=222) cohort. For this analysis patients with CDCP1 expression at or below the median score of the cohort were segregated into “low” and those with expression above the median were segregated into the “high” CDCP1 expressing group. Statistical differences between Kaplan-Meier curves were determined by Mantel-Cox test. Patients at risk in each group are indicated under each graph.

To determine the prognostic value of CDCP1 in PDAC and the proportion of patients who could potentially benefit from a CDCP1 targeted therapy, we examined its mRNA and protein expression in independent patient cohorts. To determine whether elevated CDCP1 mRNA expression associates with patient survival we analysed independent transcriptomic datasets from the TCGA and ICGC. Segregation of expression levels into quartiles demonstrated that in both cohorts, patients in the top quartile of CDCP1 expression had significantly shorter survival (p=0.0015, 43 patients; p=0.027, 67 patients) compared to those in the bottom quartile (42 and 66 patients for TCGA and ICGC cohorts respectively, Figure 1A). Additional analysis using other segregation points were consistent and showed that patients in the bottom quartile of CDCP1 expression had significantly longer survival compared to the rest of population in the TCGA cohort (p=0.0004, Figure S1A); patients in the top quartile or above the median of CDCP1 expression had significant shorter survival compared to the rest of the population in the ICGC cohort (p=0.0084 and p=0.045 respectively, Figure S1B). Analysis for associations between CDCP1 levels and clinical parameters was restricted to examination of the larger ICGC transcriptome cohort to ensure sufficient statistical power. This revealed that CDCP1 mRNA expression level is associated with tumor size (p=0.011, Figure S1C) but not with age, gender, tumor stage, vasculature invasion or lymph node positivity (data not shown).

Analysis of CDCP1 protein levels was performed by immunohistochemical examination of tissue- microarrays containing specimens from 222 cases of the Australian ICGC PDAC cohort (ICGC-PACA-AU). For controls the arrays contained normal pancreas, brain, lymph node, spleen, liver and muscle. Staining for CDCP1 was performed with antibody 4115, which detects the intracellular carboxyl-terminal of CDCP1-FL and CDCP1-CTF but cannot distinguish between the intact and cleaved receptor, and scored for both intensity (graded as 0, no staining; 1, weak; 2, median; or 3 strong) and percentage of positive cells (in 10% increments). CDCP1 expression was detected in 92% of PDAC cases but was not observed in normal pancreas, brain, lymph node, spleen, liver or muscle, with representative examples of staining shown in Figure 1B. About 10% of PDAC samples displayed strong staining, ~25% moderate and the remainder weak staining. Using these results, we performed two Kaplan-Meier survival analyses, the first comparing survival with CDCP1 staining intensity, and the second with the percentage of cells positive for CDCP1. For both analyses CDCP1 signal was initially dichotomised into scores below (low) and above (high) the median score. While no association was observed with staining intensity, high CDCP1 expression based on the percentage of positive cells (110 patients) was significantly associated with shorter overall survival compared to those assigned as low (112 patients, Figure 1C). Consistent results were obtained when the analysis was performed using other segregation points. Segregation based on quartiles showed that patients in the bottom quartile of CDCP1 expression had significantly longer survival compared to the rest of the cohort or patients in the top quartile of CDCP1 expression (p=0.0068 and p=0.017 respectively, Figure S1D). Analysis for associations between CDCP1 protein level and clinical parameters revealed no statistically significant association with age, gender, tumor size, tumor stage, vasculature invasion or lymph node positivity (data not shown).

In summary, mRNA and protein analyses demonstrate that CDCP1 is elevated in the vast majority of PDAC tumors and it is not expressed by the normal pancreas. CDCP1 expression and patient survival are inversely associated, which is suggestive that CDCP1 is functionally involved in progression of PDAC and represent an interesting target to develop anti-cancer agents for PDAC.

### Proteolytic processing of CDCP1 in PDAC cells and interaction between generated fragments

**Figure 2:**
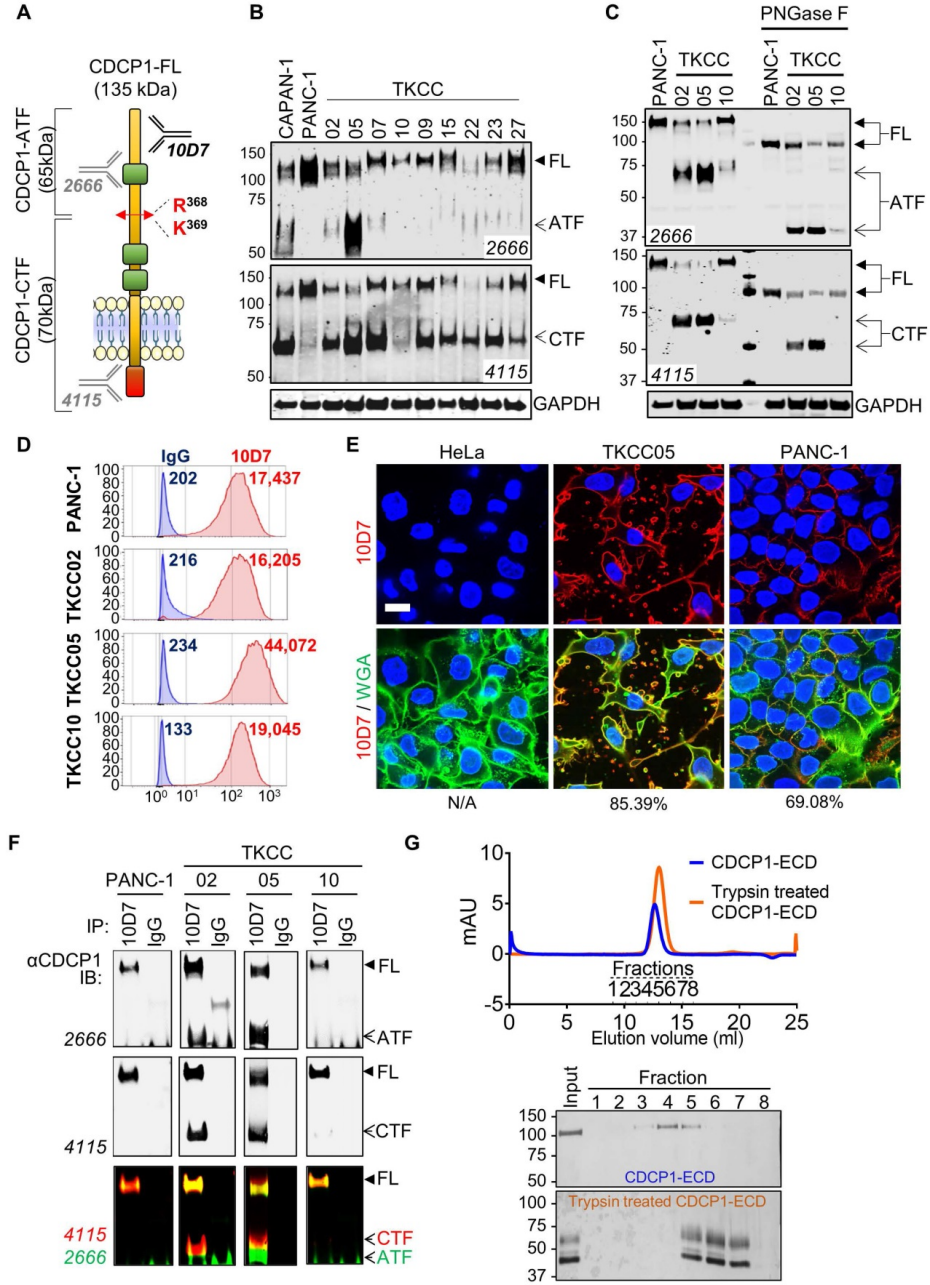
Proteolysis status of CDCP1 and interactions between CDCP1 fragments in PDAC cells. **A,** Diagram depicting structural features of CDCP1 including three extracellular CUB-like domains (green), proteolytic processing sites at R368 and K369, amino-terminal (ATF) and carboxyl-terminal (CTF) CDCP1 fragments, and binding sites of the anti-CDCP1 antibodies 10D7 (in-house), 4115 (Cell Signaling) and 2666 (R&D systems). **B,** Western blot analysis under reducing conditions of nine patient-derived PDAC cells (TKCC) and two PDAC cell lines using anti-CDCP1 antibodies 4115 and 2666, and an anti-GAPDH antibody. **C**, Western blot analysis using anti-CDCP1 antibodies 4115 and 2666, and an anti-GAPDH antibody, of PANC-1, TKCC02, TKCC05 and TKCC10 cell lysates under reduced condition before and after enzymatic deglycosylation with N-glycosidase F (PNGase F) for 1h at 37°C. **D,** Flow cytometry analysis of PANC-1, TKCC02, TKCC05 and TKCC10 cells for plasma membrane CDCP1 using antibody 10D7. **E,** Confocal microscopy imaging of HeLa, TKCC05 and PANC-1 cells after immunofluorescent staining of CDCP1 (antibody 10D7) and co-staining of nuclei and membrane with DAPI and wheat germ agglutinin-FITC (WGA), respectively. Scale bar = 20 µm. Average signal co-localization (percent) between 10D7 and WGA are indicated. **F**, Anti-CDCP1 western blot analysis with the indicated antibodies of proteins immunoprecipitated with antibody 10D7 or isotype matched control antibodies. **G,** Analysis of fractions collected by chromatography of untreated or trypsin treated CDCP1 extracellular domain (ECD). *Top,* UV/Vis spectroscopy analysis of size-exclusion chromatography. *Bottom,* Coomassie stained gel analysis of fractions collected during chromatography.

To determine the proteolysis status of CDCP1 in PDAC cells, we performed western blot analysis examining lysates from nine previously described patient-derived PDAC cells 34 and two well described PDAC cell lines, CAPAN-1 and PANC-1 40, 41. Two antibodies were employed allowing to detect the three forms of CDCP1: 135 kDa CDCP1-FL, 65 kDa CDCP1-ATF and 70 kDa CDCP1-CTF (Figure 2A). These analyses revealed that CDCP1 is expressed and cleaved to varying levels by each of the 11 PDAC cells (Figure 2B and S2A). Analysis with antibody 4115 indicated that CDCP1 is robustly cleaved in nine PDAC lines (CAPAN-1, TKCC02, TKCC05, TKCC07, TKCC09, TKCC15, TKCC22, TKCC23, TKCC27) with much lower levels of cleavage in the remaining two lines (PANC-1, TKCC10). Surprisingly, analysis with antibody 2666 indicated that 65 kDa CDCP1-ATF, which was previously identified as being shed from the cell surface 15-17, was detectable at high levels in lysates from CAPAN-1 and TKCC05, much lower levels in TKCC02, TKCC07, TKCC15, TKCC22, TKCC23 and TKCC27 (Figure 2B). CDCP1-ATF is apparent as a broad smear centred at ~65 kDa in CAPAN-1 and TKCC05 cells (Figure 2B, 2666 western blot panel). This is due to N-glycosylation because treatment of lysates from PANC-1, TKCC02, TKCC05 and TKCC10 cells with the amidase PNGase-F reduced the CDCP1-ATF molecular mass by ~25 kDa to a defined band of ~40 kDa which is close to the predicted mass of 37.9 kDa (Figure 2C). The amount of N-linked glycans on CDCP1-CTF was ~15 kDa with deglycosylation reducing its molecular mass from ~70 kDa to ~55 kDa which is also close to the predicted mass of 52.2 kDa (Figure 2C). As previously reported, CDCP1-FL contained about 40 kDa of N-linked glycans reducing from 135 kDa to ~95 kDa (Figure 2C) which is close to the predicted molecular weight of 90.1 kDa of the amino acid sequence of CDCP1 without its 29-residue signal peptide 10. These data indicate for the first time that CDCP1-ATF can be retained by PDAC cells after proteolytic cleavage, which contrasts with previous reports showing that it is shed from the cell surface after CDCP1 cleavage 15-17.

To investigate the mechanism by which CDCP1-ATF remains cell associated, we performed further analyses on PANC-1, TKCC02, TKCC05 and TKCC10 cells. These lines display variable levels of cleavage of CDCP1 (Figure 2B-C and S2A), and subcutaneous mouse xenografts exhibit histological features that are representative of the landscape of PDAC pathology (Figure S3). Flow cytometry analysis with anti-CDCP1 antibody 10D7, which binds to the extracellular domain of CDCP1 within its ATF (Fig 2A, 48), suggested that CDCP1-ATF remains cell associated via tethering to the cell surface (Figure 2D). CDCP1 signal was approximately proportional to the total level of expression of CDCP1 rather than to the level of intact CDCP1-FL.

TKCC05 cells displayed CDCP1 signal (MFI = 44,072; Figure 2D) which was more than twice the signal observed in PANC-1, TKCC02 and TKCC10 cells (MFI = 17,437; 16,205 and 19,045 respectively; Figure 2D), despite the former displaying the lowest level of intact CDCP1-FL compared the other three cells (Figure 2B-C and S2A). Immunofluorescent confocal microscopy analysis with this antibody demonstrated consistent data. TKCC05 cells stained much more strongly for cell surface CDCP1 (85.4% 10D7 signal colocalized with WGA-membrane staining) than PANC-1 cells (69.1% 10D7 signal colocalized with WGA-membrane staining) despite the former expressing much lower levels of intact CDCP1-FL, with HeLa cells, which do not express CDCP1, serving as a negative control, displaying no signal (Fig 2E).

To examine whether CDCP1-ATF remains tethered to the cell surface via binding to CDCP1-FL or CDCP1-CTF, we next performed immunoprecipitation (IP) assays with antibody 10D7 which binds to an epitope present in CDCP1-ATF. Interacting proteins are detected by western blot analysis using anti-CDCP1 antibodies 2666 and 4115. Confirming that CDCP1-ATF remains tethered to the cell surface via CDCP1, antibody 4115 detected not only uncleaved CDCP1-FL but also cleaved CDCP1-CTF in immunoprecipitates from TKCC02 and TKCC05 cells (Figure 2F). CDCP1-CTF could only have been detected if it remains linked to CDCP1-FL or CDCP1-ATF. No CDCP1-ATF signal was detected from PANC-1 or TKCC10 likely because these cells display lower levels of cleavage of CDCP1 (Figure 2B-C). Consistent with CDCP1-ATF remaining cell associated, antibody 2666 detected CDCP1-FL as well as CDCP1-ATF from TKCC02 and TKCC05 cells (Figure 2F). The interaction between CDCP1-ATF and CDCP1-FL/CTF was confirmed by the reverse IP assay using antibody 4115 (Figure S2B). In this experiment, CDCP1-FL and CDCP1-CTF were detected in immunoprecipitates from TKCC02 and TKCC05 cells, as was CDCP1-ATF (Figure S2B) which does not contain the epitope recognized by 4115 antibodies (Figure 2A). Cell-surface biotinylation experiments performed on TKCC05 cells revealed that most CDCP1 forms are located on the surface of PDAC cells (Figure S2C top, CS) compared to intracellular proteins (Int). Additionally, analysis of the levels of CDCP1 fragments found in cell lysates and conditioned media (CM) from TKCC05 cells showed that CDCP1-ATF is only detected in cell lysate (Figure S2C bottom, C) and not in CM suggesting that most CDCP1-ATF remains tethered to PDAC cells.

The existence of stable interactions between CDCP1-ATF and CDCP1-CTF was confirmed by size- exclusion chromatography experiments. Recombinant CDCP1-ECD before and after trypsin digestion was eluted as two single peaks according to UV-Vis spectroscopy analysis, at 12.63 ml for intact CDCP1- ECD and 12.85 ml for trypsin-digested CDCP1-ECD (Figure 1G, top), respectively. SDS-PAGE analysis of these peaks further confirmed that the N-terminal- CDCP1-ECD and C-terminal-CDCP1-ECD fragments products were co-eluted (Fractions 5 to 7), with an estimated molecular weight of 65 and 45 kDa, respectively, whereas the intact CDCP1-ECD was detected in fractions 3-5, with an estimated molecular mass of 110 kDa (Figure 2G bottom). To assess whether linkage of CDCP1-ATF with CDCP1-FL or CDCP1-CTF is via a disulphide bond we performed anti-CDCP1 western blot analysis with antibodies 4115 and 2666 of lysates separated under both reducing and non-reducing conditions. Analysis of PANC-1, TKCC02, TKCC05 and TKCC10 cells revealed the same protein bands observed from assays under both conditions indicating that CDCP1-ATF is not linked to CDCP1-FL or CDCP1-CTF via a disulphide bond (Fig S2D).

In summary, these data demonstrate that CDCP1 is differentially cleaved and N-glycosylated in PDAC cells producing CDCP1-ATF which remains tethered to the cell surface via non-disulfide bond interactions most likely with CDCP1-FL or CDCP1-CTF.

### Function blocking antibody 10D7 induces rapid phosphorylation, internalization and degradation of differentially cleaved CDCP1 in PDAC cells

**Figure 3:**
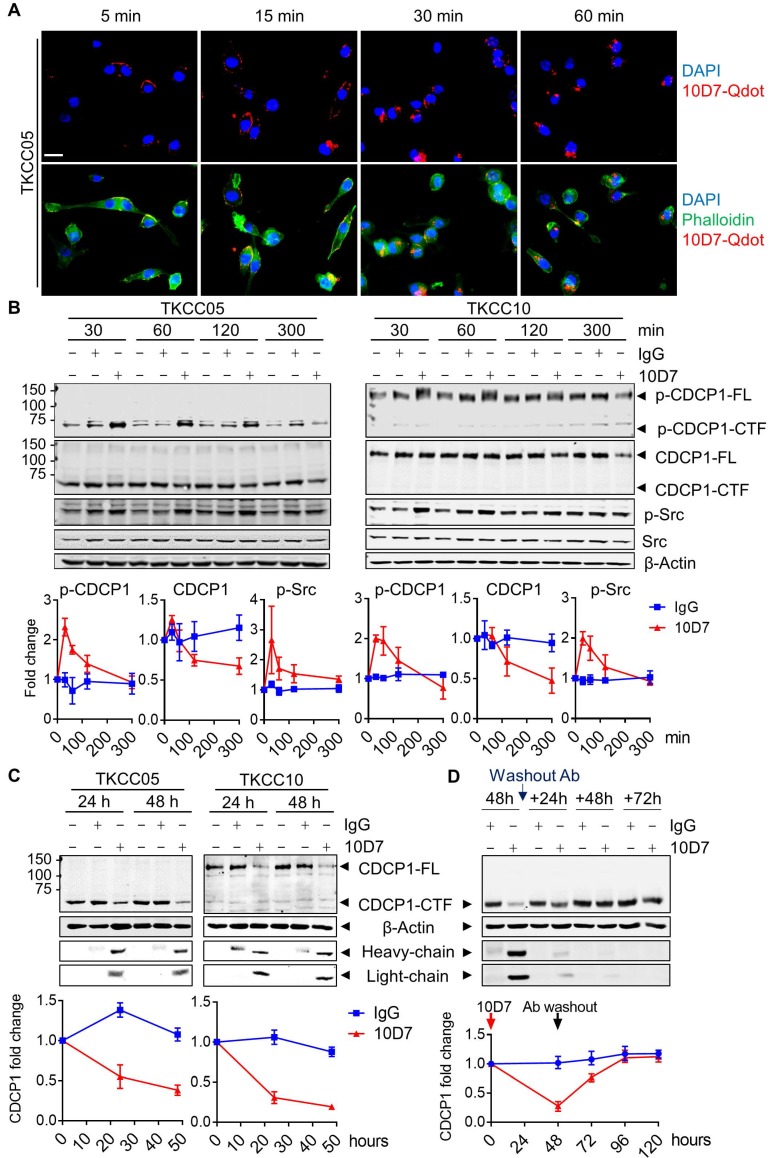
Cell binding and internalization of function blocking antibody 10D7 which induces rapid phosphorylation, internalization and degradation of differentially cleaved CDCP1 in PDAC cells. **A,** Confocal microscopy analysis of TKCC05 cells treated with fluorescently tagged antibody 10D7 (10D7-Qdot, red). After the indicated times cells were fixed then stained with phalloidin (green) and DAPI (blue) to highlight cell cytoplasm and nucleus, respectively (bottom). Specific 10D7-Qdot signal shows membrane localization then internalization of 10D7 (top). Scale bar = 25 µm. **B,** Western blot analysis of lysates from TKCC05 (*left*) and TKCC10 (*right*) cells treated for up to 300 min with antibody 10D7 or isotype matched IgG. Lysates were probed for p-CDCP1-Y734, CDCP1 (antibody 4115), p-Src-Y417, Src and β-actin. Graphs quantify changes in levels of p-CDCP1-Y734, CDCP1 and p-Src in response to 10D7 based on 3 independent experiments. **C,** Western blot analysis of lysates from TKCC05 (*left*) and TKCC10 (*right*) cells treated for longer periods with 10D7 or isotype matched IgG. Lysates were probed for CDCP1 (antibody 4115), β-actin and mouse IgG (heavy and light chains). Graphs quantify changes in levels of CDCP1 in response to 10D7 based on 3 independent experiments. **D,** Western blot analysis of lysates collected from TKCC05 cells treated for 48 h with antibody 10D7 or isotype matched IgG before antibody washout then further grown up to 72 h in normal medium. Lysates were probed for CDCP1 (antibody 4115), β-actin and mouse IgG. Graphs quantify changes in levels of CDCP1 in response to 10D7 based on 3 independent experiments. FL: Full length; CTF; carboxyl-terminal fragment; ATF: amino-terminal fragment.

Antibody 10D7 effectively blocks CDCP1 function inhibiting its roles in mouse models of vascular metastasis of prostate cancer 26 and intraperitoneal progression of ovarian cancer 13,27. At a molecular level 10D7 induces rapid Src-mediated tyrosine phosphorylation of CDCP1-Y734 (FL numbering) followed sequentially by its clustering on the surface of ovarian cancer cells then internalisation and degradation of the receptor/antibody complex in *in vitro* and mouse models 42. To assess the impact of antibody 10D7 on the different forms of CDCP1 expressed by PDAC, we performed immunofluorescent staining and western blot analysis of patient-derived PDAC cells treated with this antibody for defined periods of time. Focusing initially on TKCC05 cells, immunofluorescent microscopy revealed that despite CDCP1 being predominantly converted to CDCP1-CTF (which lacks the 10D7 binding site present within CDCP1-ATF, Fig 2A), fluorescently labelled 10D7 (10D7-Qdot) was apparent on the plasma membrane within 5 minutes of the commencement of treatment, with strong signal apparent within 15 min, and after 30 and 60 min the antibody was largely internalized, observable as intracellular puncta (Figure 3A). Western blot analysis confirmed that 10D7 induces rapid transient tyrosine phosphorylation of CDCP1-CTF in TKCC05 cells and CDCP1-FL in TKCC10 cells, while Src was also tyrosine phosphorylated in response to 10D7 (Figure 3B). Anti-CDCP1 western blot analyses indicated that by the 300 min time point that 10D7 treatments had started to reduce levels of CDCP1-CTF and CDCP1-FL (Figure 3B). Consistent data were obtained from PANC-1 and TKCC02 cells, although analysis of the latter interestingly revealed that 10D7 induces most robust phosphorylation of CDCP1-CTF not CDCP1-FL (Figure S4A). Western blot analysis also revealed that sustained treatment of PANC-1, TKCC02, TKCC05 and TKCC10 cells over 24 and 48 h with antibody 10D7 results in marked reduction in levels of both CDCP1-CTF and CDCP1-FL (Figure 3C and S4B). Removal of 10D7 resulted in gradual re-expression of CDCP1 with a return to basal levels within 24-48 h in TKCC05 cells (Figure 3D) indicating that the impact of 10D7 on CDCP1 protein levels in PDAC cells is reversible. These results collectively indicate that 10D7 is able to bind to intact CDCP1 and CDCP1-ATF/CDCP1-CTF and to induce downstream signalling, internalization of the receptor/antibody complex, and degradation of CDCP1 independently of CDCP1 cleavage status. The data are consistent with CDCP1-ATF remaining tethered to the plasma membrane to CDCP1-CTF and indicate that intact and cleaved CDCP1 can be functionally targeted with antibody 10D7 in PDAC cells.

### Antibody targeting of CDCP1 reduces cell migration and non-adherent growth, and improves chemo-responsiveness of PDAC cells *in vitro*

**Figure 4:**
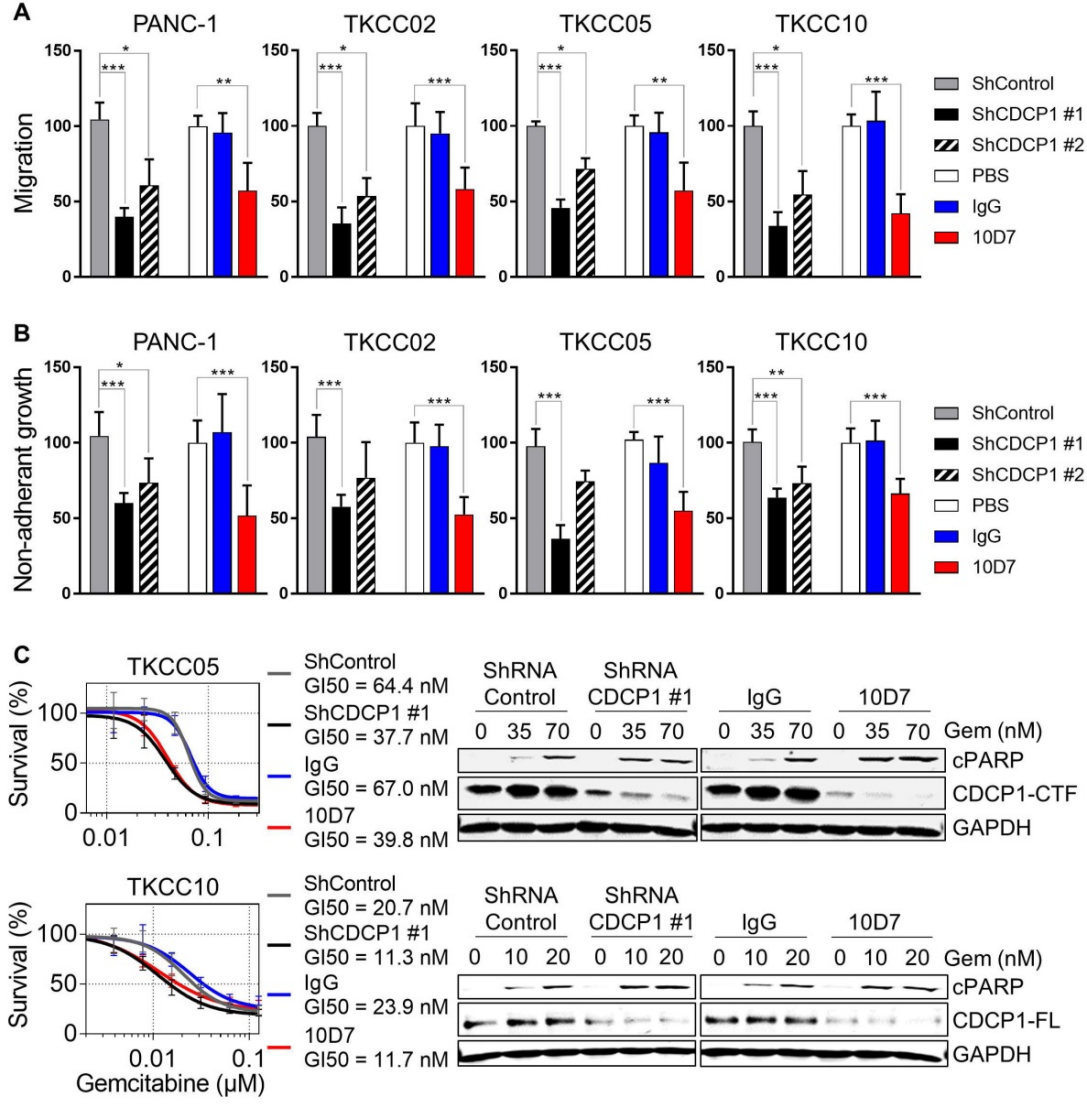
Functional consequences of CDCP1 targeting in PDAC cells *in vitro*. **A,** Transwell migration assay was performed on transduced cells (2.5 x 10^5^/well) stably expressing control shRNA, CDCP1 shRNA (two constructs) or with parental cells treated with 10D7 (5 µg/ml), isotype matched IgG (5 µg/ml) or PBS. Relative migration was determined by measurement of absorbance at 590 nm of crystal violet that was methanol extracted from stained cells. **B,** Relative spheroid growth was quantified 10 days after cell suspensions (10,000 cells/well; same condition as above) were plated in 96-well ultra-low attachment plates in serum free, growth factor restricted media. Quantification was performed by absorbance measurements at 490 nm of wells incubated with the CellTiter AQueous One Solution Reagent. **C,** *Left:* Survival analysis was performed on transduced cells stably expressing control shRNA or CDCP1 shRNA or with parental cells pre-treated with 10D7 (5 µg/ml) or isotype matched IgG (5 µg/ml) for 24 h before treatment with gemcitabine (0.02 to 500 nM) for 72 h. Relative cell survival was then determined by absorbance measurements at 490 nm of wells incubated with the CellTiter AQueous One Solution Reagent. *Right:* Western blot analysis of lysates collected from transduced cells stably expressing control shRNA or CDCP1 shRNA (construct #1) or from parental cells pre-treated with 10D7 (5 µg/ml), isotype matched IgG (5 µg/ml) treated for 24 h before treatment with 10D7 or IgG (5 µg/ml) in the presence of gemcitabine (at two concentrations close to the GI50 of each line) for 48h. Lysates were probed with antibodies against cleaved PARP (cPARP), CDCP1 (antibody 4115) and GAPDH. Statistical significance between different groups was assessed using the Kruskal-Wallis test with * p<0.05, ** p<0.01 and *** p<0.001.

To directly evaluate whether functional targeting of cleaved and intact CDCP1 can inhibit cellular processes that contribute to progression of PDAC, we employed antibody 10D7 and CDCP1-specific shRNAs in* in vitro* models assessing effects on cell migration, non-adherent growth and chemo- resistance. In the PDAC cell line PANC-1 and the patient-derived lines TKCC02, TKCC05 and TKCC10, treatment for 48 h with 10D7 reduced migration by ~50-65% which was consistent with reductions achieved by stable silencing of CDCP1 with two lentiviral shRNA constructs (Figure 4A). Western blot and flow cytometry analysis demonstrated the effectiveness of the silencing constructs at reducing total and cell surface expression of CDCP1, respectively (Figure S5A-B). Non-adherent cell growth in serum free, growth factor defined media, as a read-out for the presence of PDAC stem cell populations 43, saw a similar reduction in the number of actively dividing cell spheroids after 10 days in response to antibody 10D7 and this was also closely mimicked by stable silencing of CDCP1 (Figure 4B and S5B). Functional blockade of CDCP1 with 10D7 for 72 h also increased the *in vitro* efficacy of a chemotherapy commonly used in the treatment of PDAC, gemcitabine, halving the GI_50_ value achieved for TKCC05 and TKCC10 cells, with similar results obtained from silencing of CDCP1 (Figure 4C, left). Interestingly, the improved efficacy of gemcitabine caused by 10D7 was accompanied by a marked increase in cell death as evidenced by increasing levels of cleaved PARP even though CDCP1 had been largely degraded in response to continuous treatment with the antibody for 72 h (Figure 4C, right). These data indicate that independent of the state of CDCP1 cleavage, 10D7 is effective at targeting PDAC cells *in vitro*.

### PET imaging based detection of PDAC cells *in vivo* using 10D7 antibody

**Figure 5:**
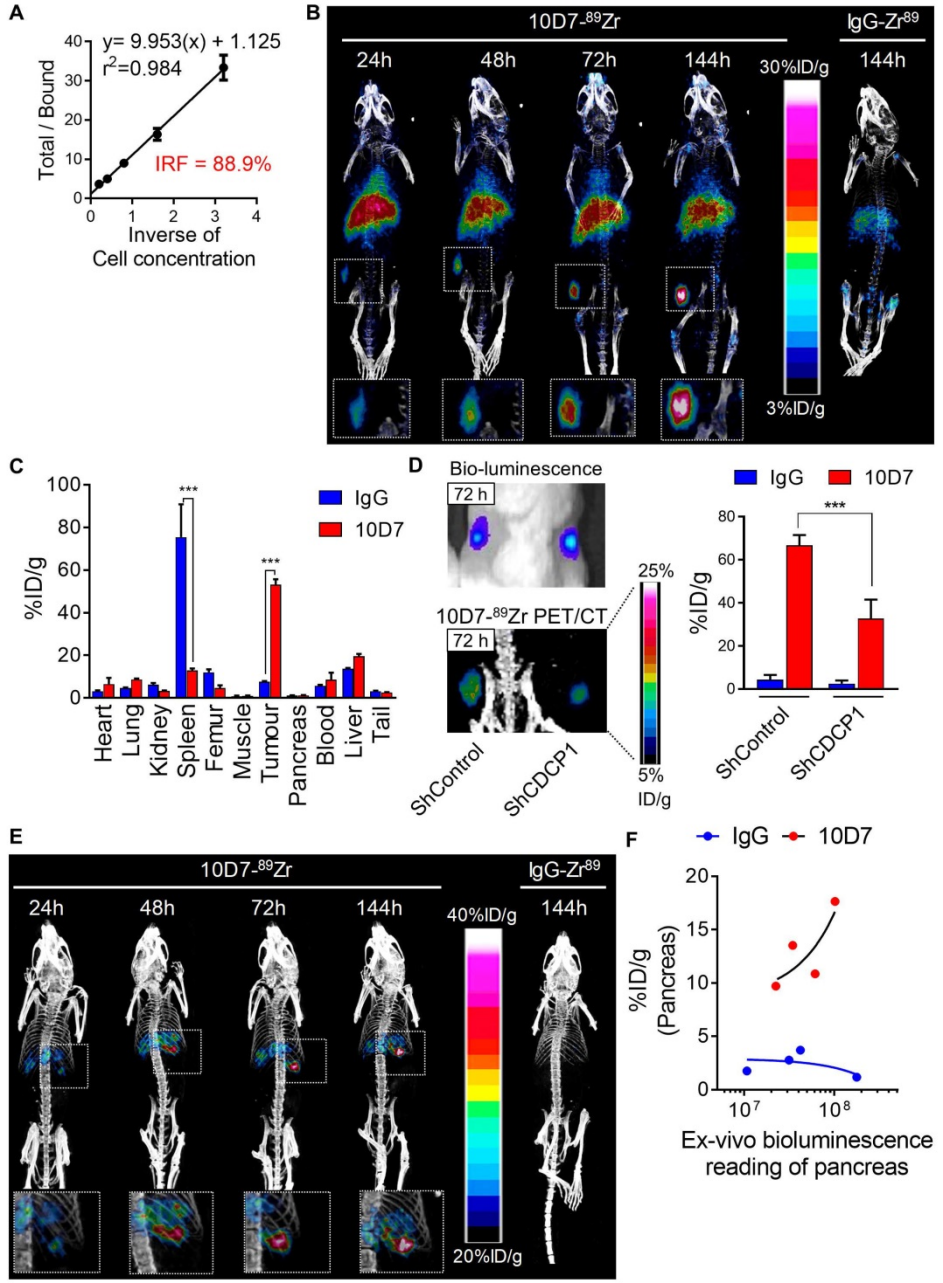
10D7 antibody detects PDAC cells *in vivo*. **A,** Lindmo plot showing binding of 10D7-^89^Zr to an increasing number of CDCP1-positive TKCC05 cells. **B,** Representative PET-CT images of NSG mice carrying subcutaneous TKCC05 cell tumors. 10D7-^89^Zr-and IgG1κ-^89^Zr were injected intravenously two weeks after tumor cell inoculation, and imaging performed 24, 48, 72 and 144 h later. **C,** Quantitative bio-distribution analysis of 10D7-^89^Zr and IgG1κ-^89^Zr 144 h post injection (n = 3). **D,** *Left:* Bioluminescence imaging (top) and PET-CT imaging with 10D7-^89^Zr as the contrast agent (bottom) of TKCC05-shCDCP1 and TKCC05-shControl cell xenografts. *Right:* Quantitative distribution analysis of 10D7-^89^Zr and IgG1κ-^89^Zr 144 h post injection (n = 3) in TKCC05-shCDCP1 compared with TKCC05-shControl cell xenografts. Statistical significance between different groups was performed using a two-way ANOVA test with *** p<0.001. **E,** Representative PET-CT images of NSG mice carrying intra-pancreas TKCC05 cell tumors. 10D7-^89^Zr-and IgG1κ-^89^Zr were injected intravenously four weeks after tumor cell inoculation, and imaging performed 24, 48, 72 and 144 h later. **F,** Plot of the *ex vivo* level of radioactivity (%ID/g) versus bioluminescence of pancreas from mice which received either 10D7-^89^Zr or IgG1κ-^89^Zr measured 144 h post injection.

To evaluate whether the ability of 10D7 to disrupt cellular processes that promote PDAC *in vitro* can be harnessed to target this cancer *in vivo*, we first evaluated the capacity of this antibody to detect PDAC xenografts in mice. This was first performed by immuno-PET/CT imaging of mice bearing subcutaneous xenografts of TKCC05 cells. Antibody 10D7 was efficiently conjugated with the positron emitting radionuclide ^89^Zr as indicated by Lindmo assay analysis demonstrating an intact 10D7-^89^Zr IRF of 88.9% (Figure 5A). Imaging was performed on relatively small (~100 mm^3^) tumors two weeks after subcutaneous injection of TKCC05 cells into host mice. Two randomised groups of mice (n=3/group) were administered intravenous 10D7-^89^Zr or IgG-^89^Zr (average dose 1.5 MBq/mouse) and PET/CT imaging was performed at 24, 48, 72 and 144 h time points. A strong time-dependent accumulation in tumors of 10D7-^89^Zr but not IgG-^89^Zr was observed with signal clearly visible at 24 h in tumors of all mice administered 10D7-Zr^89^ (Figure 5B). Experimental endpoint radioactivity biodistribution analysis confirmed that 10D7-^89^Zr, in contrast with IgG-^89^Zr, predominantly accumulated in tumors (53.1%ID/g versus 7.6%ID/g; Figure 5C). Non-specific accumulation of IgG-^89^Zr was particularly strong in spleen (76%ID/g for IgG-^89^Zr versus 13%ID/g for 10D7-^89^Zr), and both 10D7-^89^Zr and IgG-^89^Zr accumulated non-specifically at lower levels in liver and femur (Figure 5C), sites which are commonly observed in mouse models 44.

To confirm that 10D7-^89^Zr accumulation in PDAC tumors is dependent on CDCP1 expression, we compared the signal obtained from subcutaneous tumors of TKCC05 cells stably transduced with CDCP1 silencing or control lentiviral constructs. CDCP1 silenced and control tumors in the flanks of mice grew at the same rates (Figure 5D, left upper panel) and displayed 10D7-^89^Zr signal in tumors in proportion to the level of expression of CDCP1 (37.7%ID/g vs 66.7%ID/g, Figure 4D left lower and right panels). As expected, minimal signal was observed in tumors of mice administered IgG-^89^Zr (Figure 5D, right panel).

We also assessed the efficacy of 10D7-^89^Zr against orthotopic xenografts of TKCC05 cells. As for subcutaneous tumors, there was a time dependent accumulation of 10D7-^89^Zr in intra-pancreas tumors with little evidence of localization of control IgG-^89^Zr at this site (Figure 5E). To ensure that this lack of accumulation of IgG-^89^Zr was not because these mice had much lower tumor burden, we measured the *ex vivo* bioluminescence of whole pancreas plotting this value against radiation levels from pancreas of mice injected with 10D7-^89^Zr or IgG-^89^Zr. For the mice which received 10D7-^89^Zr, the level of radioactivity measured is proportional to the level of bioluminescence demonstrating that the level of accumulation of 10D7-^89^Zr is dependent on tumor size (Figure 5F, red dots). No such correlation was observed for the mice which received IgG1κ-^89^Zr (Figure 5F, blue dots) demonstrating that the accumulation of radioactivity in tumor was specifically due to the binding of 10D7 to CDCP1-expressing cells and not to unspecific accumulation of antibodies within tumors related to tumor size. Overall these data demonstrate the ability of antibody 10D7 to selectively target CDCP1 expressing PDAC tumors *in vivo*.

### Antibody targeting of CDCP1 reduces tumor burden and improves gemcitabine efficacy *in vivo*

**Figure 6:**
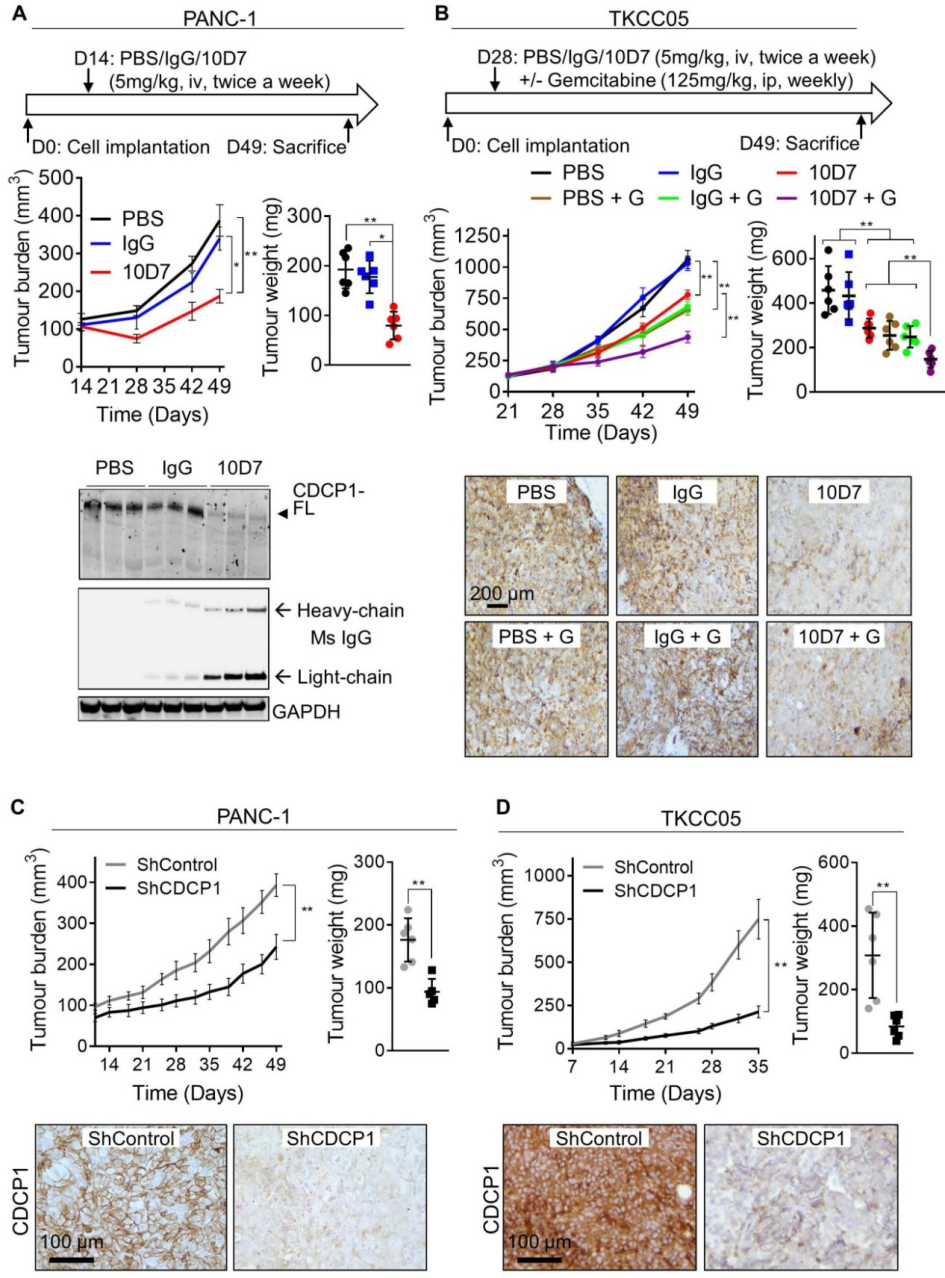
Functional targeting of CDCP1 reduces tumor burden and improves gemcitabine efficacy *in vivo*. **A.** Effect of antibody targeting of CDCP1 on PANC-1 cell xenograft growth. *Top*, Two weeks after subcutaneous inoculation of PANC-1 cells (average tumor size ~100 mm^3^) mice (6/group) were randomized and treated i.v. twice a week with PBS, 10D7 (5 mg/kg) or IgG (5 mg/kg). *Middle left*, Graph of tumor volume measured weekly by calliper. *Middle right*, Graph of tumor weight at experimental end-point after 7 weeks of growth. *Bottom,* Western blot analysis of lysates collected from representative PANC-1 cell xenografts. Antibodies were against CDCP1 (4115), mouse IgG and GAPDH. **B,** Effect of antibody targeting of CDCP1 on TKCC05 cell xenograft growth in combination with gemcitabine chemotherapy. *Top*, Three weeks after subcutaneous inoculations of TKCC05 cells, mice were randomized and treated i.v. twice a week with PBS (n=12), 10D7 (n=12, 5 mg/kg) or IgG (n=12, 5 mg/kg). Half of the mice in each group also received gemcitabine i.p. treatments (125 mg/kg weekly the day after antibody treatment). *Middle left*, Graph of tumor volume measured weekly by calliper. *Middle right*, Graph of tumor weight at end-point. *Bottom,* Representative anti-CDCP1 (antibody 4115) stained sections from recovered TKCC05 cell xenografts. **C and D,** Impact of CDCP1 silencing on growth of subcutaneous xenografts of PANC-1 (C) and TKCC05 (D) cells stably expressing ShRNA control (ShControl) or ShRNA CDCP1 (ShCDCP1). *Top left*, Graph of tumor volume measured weekly by calliper. *Top right*, Graph of tumor weight at end-point. *Bottom*, Representative images of immunohistochemical analysis of CDCP1 expression (antibody 4115) in xenografts. Statistical significance has been determined by Mann-Whitney test between indicated groups with * p<0.05, ** p<0.01 and *** p<0.001.

We next examined whether the ability of antibody 10D7 to disrupt PDAC cells *in vitro* and detect PDAC tumors *in vivo*, can be harnessed to inhibit growth of established subcutaneous xenografts in mice of luciferase expressing PANC-1 and TKCC05 cells. Twice weekly treatments with 10D7 (5 mg/kg) for 5 weeks significantly slowed growth of PANC-1 tumors and reduced end-point tumor weight by ~60% compared with treatments with isotype matched IgG and PBS (Figure 6A, upper panels). Consistent with results from *in vitro* assays (Figure 4C), 10D7 treatments markedly reduced CDCP1 levels in PANC-1 xenografts as assessed by immunohistochemical (Figure S6A) and western blot (Figure 6A, lower panel and S6B) analysis. For TKCC05 cell xenografts, which have a faster growth rate than PANC-1 cell xenografts, we examined whether antibody 10D7 improves the efficacy *in vivo* of gemcitabine. Treatment with 10D7 (5 mg/kg) every four days in combination with two weekly treatments with the chemotherapy (125 mg/kg) significantly slowed the growth of subcutaneous xenografts of TKCC05 cells (Figure 5B, upper left panel). At end-point the combination resulted in ~30% reduction in tumor weight compared with treatments with the single agents or PBS (Figure 5B, upper right panel). As seen in PANC-1 xenografts, 10D7 treatments reduced the level of CDCP1 expression by TKCC05 cell xenografts (Figure 6B, lower panel and S6B). These data are consistent with findings from *in vitro* assays showing that antibody 10D7 has anti-PDAC effects and that it results in degradation of CDCP1 (Figure 3). Also, consistent with effects on tumor burden caused by 10D7, stable silencing of CDCP1 markedly reduced tumor burden of subcutaneous xenografts of PANC-1 and TKCC05 cells compared with xenografts of these cells stably transduced with scramble control vectors (Figure 6C-D and S6C-D).

### Antibody 10D7 is effective for specific cytotoxin delivery to PDAC cells *in vitro* and *in vivo*

**Figure 7:**
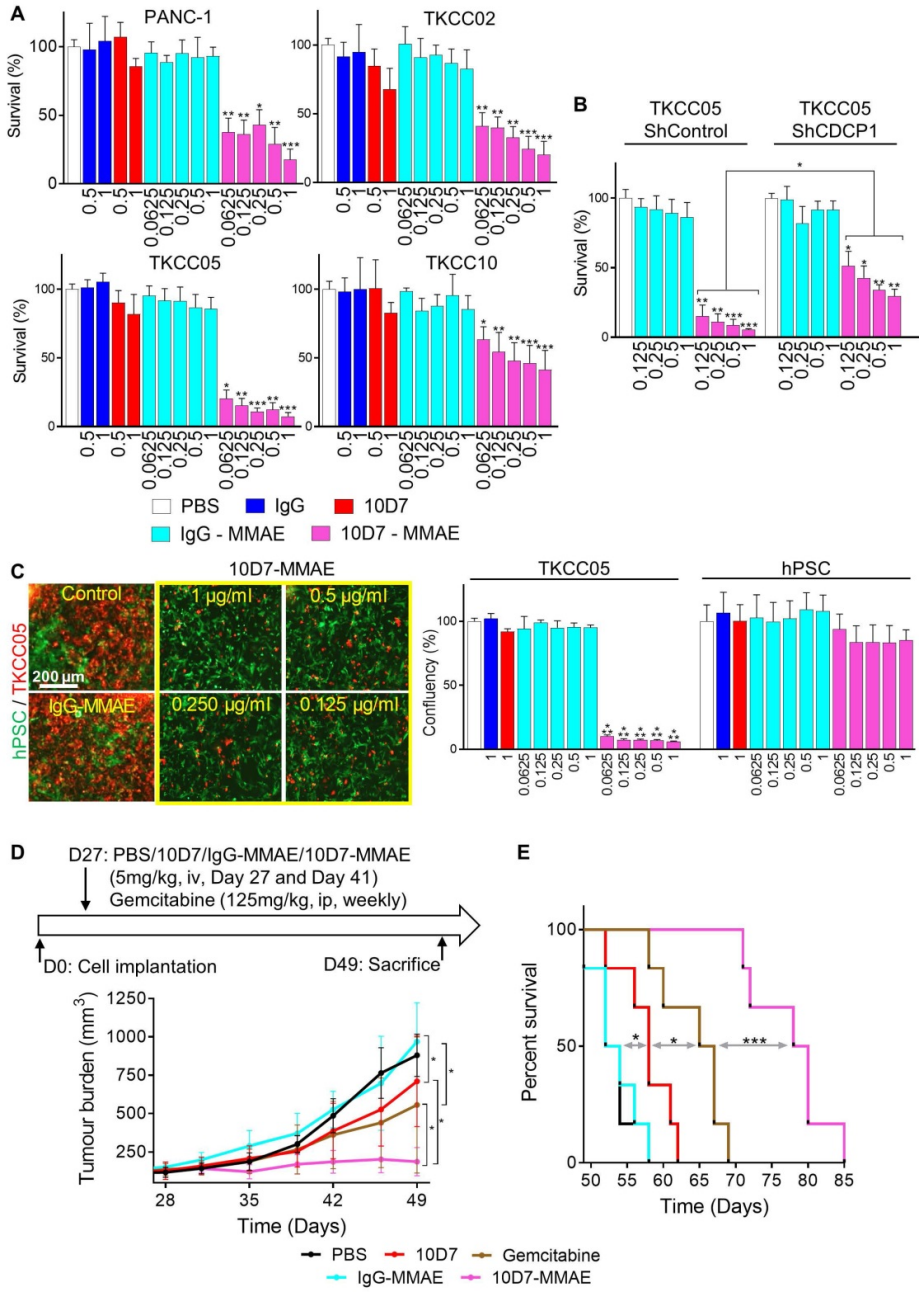
Antibody 10D7 is effective for specific cytotoxin delivery to PDAC cells *in vitro* and *in vivo*. **A,** Relative survival of PANC-1, TKCC02, TKCC05 and TKCC10 cells treated for 12 h with 10D7-MMAE or IgG-MMAE (0.0625, 0.125, 0.25, 0.5 and 1 µg/ml) or 10D7 or IgG (0.5 and 1 µg/ml) then grown for a further 72 h in complete medium. Quantification was performed by absorbance measurements at 490 nm of wells incubated with the CellTiter AQueous One Solution Reagent. Data are presented as mean +/- SD from 3 independent experiments. **B,** Relative survival of TKCC05-shControl and TKCC05-shCDCP1-1 cells treated as above. Data are presented as mean +/- SD from 3 independent experiments. **C,** *Left*, TKCC05 cells expressing monomeric Kusabira-Orange 2 (mKO2; red) co-cultured with GFP-expressing normal human pancreatic stellate cells (hPSC; green) treated as above. *Right*, Graph of survival of TKCC05 and hPSC cells quantified from the confluency area for each cell type from fluorescent microscopy images from the red and green channels, respectively. Data are presented as mean +/- SD from 3 independent experiments.** D,** Effect on growth of antibody-mediated cytotoxin delivery to CDCP1 expressed by subcutaneous TKCC05 cell xenograft. *Top*, Day 27 after inoculation of TKCC05 cells, mice (6/group) were randomized and treated on that day and day 41 i.v. with PBS, 10D7 (5 mg/kg), IgG (5 mg/kg), 10D7-MMAE (5 mg/kg) or, IgG-MMAE (5 mg/kg), or on day 27, 34, 41 and 48 with i.p. gemcitabine (125 mg/kg). *Bottom*, Graph of tumor volume measured weekly using callipers until day 49 when the first mice in the control groups required euthanasia due to disease burden. **E,** Kaplan-Meier survival curve of mice in each treatment group from D. Statistical significance in comparison to control group (PBS) was determined by the Kruskal-Wallis test with * p<0.05, ** p<0.01 and *** p<0.001. The Mann-Whitney test has been used when two-groups are compared (B and D). Statistical significance of the survival analysis was performed using Log-rank Gehran-Breslow Wilcoxon Chi^2^ test.

To assess the ability of 10D7 to deliver cytotoxic payloads to PDAC, we labelled it and isotype matched control IgG with the highly potent toxin MMAE via a link incorporating a lysosomal protease cleavage site that promotes intracellular release of the toxin and cell death (45). The generated antibody-drug conjugate (ADC), 10D7-MMAE, has an average DAR of 4.5 to 4.7, and retains the functional ability to induce phosphorylation of CDCP1 and Src 48. 10D7-MMAE significantly reduced survival *in vitro* in a dose-dependent manner of PANC-1, TKCC02, TKCC05 and TKCC10 cells compared with IgG, 10D7 and IgG-MMAE controls (Figure 7A). Of note, comparing the naked 10D7 antibody and 10D7-MMAE, at a concentration of 1 µg/ml, survival of PANC-1, TKCC02, TKCC5 and TKCC10 cells reduced, respectively, from ~85% to ~20%, ~75% to ~25%, ~85% to ~10% and ~80% to ~50% (Figure 7A). The relative resistance of TKCC10 cells to 10D7-MMAE may relate to its lower level of CDCP1 expression or an as yet unidentified cellular mechanism by which this cell type processes 10D7-MMAE with lower efficiency. Demonstrating that 10D7-MMAE is mediating its anti-survival effects via binding to CDCP1, TKCC05 cells with reduced levels of CDCP1 (using lentiviral construct shCDCP1-1; Fig S5A and B) displayed much lower responsiveness to 10D7- MMAE than these cells stably transduced with a scramble control silencing vector (Figure 7B). The selectivity of 10D7-MMAE for CDCP1 expressing cells was confirmed by treatment of co-cultures of CDCP1 expressing TKCC05 cells and non-expressing hPSCs (Fig 7C, left; red and green cells, respectively). Whereas the hPSCs were unresponsive to 10D7-MMAE, TKCC05 cells were very sensitive to this agent (Figure 7C, right).

Finally, we evaluated whether the PDAC targeting ability of antibody 10D7 can be harnessed to effectively deliver the cytotoxin MMAE to PDAC *in vivo*. On day 27 and 41 after cell injections, mice with established subcutaneous TKCC05 cell xenografts were treated with 10D7-MMAE, the naked 10D7 antibody or IgG labelled with MMAE (IgG-MMAE), or on day 27, 34, 41 and 48 with gemcitabine. Of significance, 10D7-MMAE markedly inhibited tumor growth (Figure 7D) and significantly extended survival of xenografted mice (Figure 7E and S6E) in comparison with the other treatments.

## Discussion

The key finding from this study is that antibody- mediated targeting of the receptor CDCP1 is an effective approach to deliver cytotoxins to kill PDAC cells *in vitro* and *in vivo*, and to deliver positron-emitting radionuclides for PET imaging of PDAC xenografts in mice. Our finding that CDCP1 expression is elevated in the vast majority of PDAC patient tumors, but not expressed by normal pancreas or other major organs, supports CDCP1 as a target for delivery of agents that could assist in the prognostication and treatment of PDAC. Our PDAC patient expression data add considerably to a consistent previously reported study 25, because we have demonstrated association between poor patient prognosis and elevated CDCP1 expression at both mRNA and protein levels analyzing three patient cohorts each containing more than one hundred patient samples.

Interestingly, we have also identified that CDCP1 is differentially proteolytically processed in patient-derived PDAC cells and PDAC cell lines. In some of these cells it is predominantly present as CDCP1-FL, the intact 135 kDa receptor, while in others it is predominantly cleaved generating the 70 kDa membrane spanning CTF and the 65 kDa ATF. While the ATF has previously been shown to be shed from the surface of prostate cancer cells *in vitro* 15,16 and to be present in the serum of colorectal cancer patients 17, this study for the first time demonstrated that this fragment of CDCP1 could be retained on the cell surface. Plasma membrane retention was directly demonstrated by flow cytometry analysis, immunoprecipitation and cell fractionation experiments using antibodies 10D7 and 2666 that bind to CDCP1-FL and -ATF and antibody 4115 which binds to CDCP1-FL and -CTF. These antibodies were as effective at detecting CDCP1 in cells that predominantly express CDCP1-FL as those that predominantly cleave this receptor. Additionally, we also observed that CDCP1 glycosylation status is variable in different PDAC cell lines. Currently, the importance of this variability in regulating interactions between the different CDCP1 fragments as well as the binding of antibody to CDCP1 is not known.

While the exact mechanism by which CDCP1- ATF remains tethered to CDCP1 is yet to be determined, our data indicate that it occurs via a non-disulfide bond linkage, most likely to CDCP1- CTF. In *in vitro* experiments using trypsin treated recombinant CDCP1-ECD, N-terminal-CDCP1-ECD interacts with C-terminal-CDCP1-ECD suggesting that CDCP1-ATF could interact with the extracellular portion of CDCP1-CTF. However, we cannot exclude possible binding of CDCP1-ATF to intact CDCP1-FL. In future studies it will be interesting to determine the molecular and biological functions of tethering CDCP1-ATF to CDCP1. In particular, the binding of CDCP1-ATF to CDCP1-FL and/or -CTF could impact CDCP1 dimerization, which has been demonstrated previously 8, or CDCP1 interactions with partners that are at least partly regulated by CDCP1 proteolysis including Src family kinases and acyl CoA-synthetase ligase 8, 29.

Although CDCP1 is commonly cleaved in patient-derived PDAC cells, our data suggest that its proteolysis is not a significant contributor to the PDAC cell movement, resistance to chemotherapy and primary tumor growth that we observed in our assays. We make this proposal because we observed that silencing CDCP1 expression was largely as effective as its antibody-mediated disruption at slowing cell migration and non-adherent growth *in vitro*. Also, xenograft growth *in vivo* and gemcitabine efficacy *in vitro* and *in vivo* appeared to be independent of whether CDCP1 was predominantly cleaved or intact. This contrasts with other reports where CDCP1 cleavage was important in promoting cancer including in chick embryo and mouse models of vascular metastasis of prostate cancer 6 and migration of breast cancer cells *in vitro* 8. Importantly, it is not possible based on our data to exclude that CDCP1 cleavage is not important in other processes that drive the aggressiveness of PDAC including dissemination to secondary sites or interactions with stromal or immune components.

Another important finding from this study is that the cleavage status CDCP1 did not appear to impact the ability of antibody 10D7 to deliver imaging and cytotoxic agents *in vivo* to PDAC cells. Also of note, *in vivo* accumulation of radiolabelled 10D7 was predominantly in xenografts, with limited signal from normal tissues. Consistent with this high tumor selectivity, we observed that a “weaponized” form of 10D7, conjugated with the highly toxic agent MMAE, had significant ability to impede PDAC xenograft growth and improve mouse survival with no evidence of off-target effects. Nevertheless, because PDAC has an extraordinarily dense fibrotic stroma that impedes tumor perfusion and delivery of anticancer agents 31 it is possible that CDCP1-targeted agents could fail in these patients. To address this potential issue CDCP1-targeted therapies could be combined with agents that deplete matrix components to improve tumor perfusion and payload delivery, such as the recently described CSG peptide 46.

In summary, our data suggest that CDCP1 has clinical potential as a target for delivery of agents PET imaging and/or treatment of PDAC. Further work is required to determine the importance of cleavage of CDCP1 to progression of PDAC and whether this occurs in patient tumors and will negatively impact on the efficacy of agents that target the ATF of CDCP1 for delivery of positron-emitting radionuclides or therapeutic toxic agents.

## Supplementary Material

Supplementary materials and methods, figures, table.
