# Supplementary material for: Effective targeting of intact and proteolysed CDCP1 for imaging and treatment of pancreatic ductal adenocarcinoma
Source: Theranostics. 2020 Mar 4;10(9):4116–33. doi: 10.7150/thno.43589 (PMC7086361; doi:10.7150/thno.43589)
Supplement: Supplementary file 1 — Supplementary materials and methods, figures, table. [file thnov10p4116s1.pdf]

Supplementary MATERIALS and METHODS

Chemicals and reagents

All chemical reagents were purchased from Sigma-Aldrich (Castle Hill, Australia) except where noted and all tissue culture reagents were from Thermo Fisher Scientific (Mulgrave, Australia). The previously described mouse monoclonal anti-CDCP1 antibody 10D7 [24] and mouse monoclonal control isotype $IgG_1\kappa$ antibody were purified from hybridoma culture supernatant by The Walter and Eliza Hall Institute of Medical Research antibody facility (Parkville, Australia). Mouse anti-CDCP1 antibody 2666 was from R&D Systems (In vitro Technologies, Noble Park, Australia). Rabbit anti-CDCP1 (#4115), rabbit anti-p-CDCP1-Y734 (#9050), mouse anti-Src (#2110), rabbit anti-p-Src-Y416 (#2101), mouse anti-PCNA (PC10), mouse anti-GAPDH (D4C6R), anti-rabbit IgG (H+L) DyLight™ 680 conjugate (#5366) and anti-mouse IgG (H+L) DyLight™ 800 4X PEG Conjugate (#5257) were from Cell Signaling Technologies (Genesearch, Arundel, Australia). Rabbit anti-Cleaved-PARP antibody (ABC26) was from Merck (Macquarie Park, Australia). Rabbit anti-Cystatin-C antibody (ab109508) was from Abcam (Melbourne, Australia). Goat anti-rabbit IgG (H+L) cross-adsorbed Alexa Fluor® 594, goat anti-mouse IgG (H+L) Alexa Fluor® 647, propidium iodide (PI), Qdot 625 fluorescent probe labelling kit, wheat germ agglutinin (WGA) Alexa Fluor 488 conjugate, Alexa Fluor 488 phalloidin and DAPI solution were from Thermo Fisher Scientific and Complete EDTA-free protease inhibitor mixture was from Sigma-Aldrich. CellTiter AQueous One Solution Reagent was from Promega (Hawthorn East, Australia) and specialized blocking reagents for western blot and flow cytometry were from Rigby Laboratories (Kalbar, Australia). The maleimide activated drug linker, incorporating monomethyl-auristatin E (MMAE) for generation of antibody-drug conjugates, was maleimidocaproyl-valine-citrulline-p-aminobenzoyloxycarbonyl-MMAE (MC-VC-PAB-MMAE) and was purchased from Levena (San Diego, CA).

Lysate preparation, immunoprecipitation and Western blot analysis

Cells were lysed in RIPA buffer containing EDTA-free Complete protease inhibitor (1x), sodium vanadate (2 mM) and sodium fluoride (10 mM). Snap frozen tissues (2-3 mm^3) were lysed in the presence of Lysing Matrix D ceramic particles (MP Biomedicals, Seven Hills, Australia) on a FastPrep-24 tissue disruptor (MP Biomedicals). Lysates were homogenized by passing the samples through 26-G needles and cleared by centrifugation at 14,000 g and 4°C for 30 min. Protein concentration was quantified by micro-bicinchoninic acid assay (Thermo Fisher Scientific). For protein deglycosylation, reduced lysates were treated with

PNGase F from New England Biolabs (Genesearch) for 1h at 37°C. Immunoprecipitation (IP) was performed using antibodies 10D7 and 4115 and, respectively, Protein-G and Protein-A agarose beads (Sigma-Aldrich). For antibody 10D7 IP, the lysis buffer was 1% CHAPS in PBS containing 1x EDTA-free protease inhibitors cocktail while antibody 4115 IP was performed as previously described [17]. Lysates (40 µg for cells and 80µg for tissues) or immunoprecipitates were separated by SDS-PAGE under reducing conditions (except where noted), transferred onto nitrocellulose membranes, and blocked in fish gelatin blocking buffer (3% w/v in PBS). Membranes were incubated with primary antibodies diluted in blocking buffer overnight at 4°C, washed with PBS containing 0.1% Tween 20, and then incubated with appropriate secondary antibody. Signals were detected using an Odyssey Imaging System and software (LI-COR Biosciences, Millennium Science, Mulgrave, Australia). Densitometry analyses were performed using ImageJ software [37].

Cell surface biotinylation

Cell surface proteins were isolated using cell impermeant EZ-link NHS-SS-biotin (1.22 mg/mL) as described previously [47]. Briefly, TKCC05 cells at 50% confluence stably expressing ShCDCP1#1 or ShControl were washed with PBS and biotinylated for 1 h at 4°C. Cells were washed with PBS and whole cell lysates collected in lysis buffer (1% (v/v) Triton X-100, 50 mM Tris/HCl (pH 7.4), 150 mM NaCl and 1× protease inhibitor cocktail). After removal of cellular debris by centrifugation (3000 rpm for 10 min at 4°C), lysates were incubated with streptavidin beads (Pierce) for 30 min at 4°C with gentle agitation. Biotinylated cell surface proteins immobilised on streptavidin beads were pelleted by centrifugation (3000 rpm for 5 min at 4°C) and together with intracellular proteins present in the supernatant were examined by Western blot analysis.

Analysis of trypsin cleaved CDCP1

The recombinant extracellular domain (**ECD**) of CDCP1 was expressed and purified from HEK293 cells as previously described [18]. Purified CDCP1-ECD resuspended in HEPES buffered saline (0.5 mg/ml) was treated with trypsin (2 µg/ml) at room temperature for 30 min. The reaction was stopped by addition of Phenylmethanesulfonyl fluoride (PMSF) to a final concentration of 1 mM followed by incubation for 30 min on ice. Reaction products and untreated CDCP1-ECD were separated by size-exclusion chromatography using a Superdex 200 100/300 GL column (GE Healthcare, Chicago, IL) using HBS with 1 mM PMSF as a running buffer. Collected fractions were analysed by UV/Vis spectroscopy and SDS-PAGE.

Determination of number of cell-surface CDCP1 molecules and binding of 10D7 on PDAC cells by flow cytometry

To quantify the number of cell surface CDCP1 receptors, flow cytometry analyses were performed using the phycoerythrin (PE) tagged anti-CDCP1 antibody CD318-PE (BioLegend, Karrinyup, Australia) and a standard curve generated using dilutions of a known concentration of PE-Quantibrite Beads (BD Biosciences, Hamilton, Australia). Cells detached non-enzymatically were blocked in PBS/0.5% BSA (30 minutes; 4°C) before incubation of known numbers of cells with antibody CD318-PE (0.25, 0.5 and 1 μM) which were then analysed by flow cytometry using an Accuri C6 cytometer (BD Biosciences). This identified a saturating concentration of CD318-PE molecules per cell and the corresponding MFI value was used to interpolate the number of CDCP1 receptors per cell from a standard curve of the $\log_{10}$ values for the number of PE molecules per Quantibrite bead against the $\log_{10}$ of the corresponding MFI values.

To assess levels of cell surface CDCP1, adherent cells detached non-enzymatically were blocked in PBS/0.5% BSA (30 minutes; 4°C) before incubation with 10D7 or isotype control IgG (5μg/ ml, 1h at 4°C). The cells were then washed with PBS then incubated with an APC-conjugated anti-mouse secondary antibody (BioLegend) in PBS/0.5% BSA (30 min; 4°C). After PBS washes, cells were stained with PI to assess cell death which occurred during staining and analysed on a Fortessa X-20 flow cytometer.

Spinning-disk confocal microscopy

Antibody 10D7 was labelled with a fluorescent Qdot 625 probe by following the instructions of the manufacturer. TKCC05 cells were grown on poly-Lysine treated 1 μm chamber slides (DKSH, Hallam, Australia) until 70% confluent then incubated with 10D7-Qdot. After 5, 10 and 120 minutes cells were fixed with 4% paraformaldehyde for 15 min at room temperature (RT), washed with PBS, then incubated with DAPI to highlight cell nuclei and Alexa Fluor 488 phalloidin to highlight cell cytoplasm (30 min at RT). Imaging was performed on a Spectral Spinning Disc Confocal microscope (Nikon Australia, St Kilda, Australia).

Antibody-drug conjugation

To conjugate 10D7 and IgG1κ with MMAE, antibody inter-chain disulfides were first partially reduced using DTT (10 nM, 15 min, 37°C) to generate free thiols, which were

reacted with excess maleimide activated MC-VC-PAB-MMAE in 10% DSMO for 2 h at 37°C [39,40]. Reaction impurities were removed from crude 10D7-MMAE and IgG-MMAE recation mixtures by filtering through Amicon Ultra Centrifugal Filters (Sigma-Aldrich). The drug-antibody ratio (DAR) of purified labelled antibodies was determined by reverse phase LC/MS analysis of separated light and heavy chains as reported [41,42]. Average DAR was of 4.5 to 4.7.

Cell migration, non-adherent growth and survival assays

To assess migration, cells (2.5×10^4) were seeded in serum free media into the top chamber of 24-well Transwell chambers containing a polycarbonate nucleopore membrane (8 μm pores; Corning, Crown Scientific, Minto, Australia). Cells were then treated with 10D7 (1 or 5 μg/ml), isotype matched IgG (5 μg/ml) or PBS. The chemoattractant in the bottom well was serum containing media. After 48 h migrated cells were fixed with methanol, stained with 0.2% crystal violet and imaged by microscopy. For quantification, crystal violet was extracted with methanol and absorbance at 590 nm was measured using a POLARstar Omega plate reader (BMG Labtech, Mornington, Australia).

To measure non-adherent spheroid growth, cell suspensions (10,000/well) were plated in 96-well ultra-low attachment plates (Corning) in serum free, growth factor restricted media [14] supplemented with 10D7 (5 μg/ml), isotype matched IgG (5 μg/ml) or PBS. To allow longer-term cell proliferation, 100 μl of medium was replaced with fresh medium every 3 days. Relative spheroid growth was quantified after 10 days by adding the CellTiter AQueous One Solution Reagent to wells and measuring absorbance at 490 nm using a POLARstar Omega plate reader.

For survival assays, cells (5,000/well) were plated in 96-well plates and allowed to attach for 24h. Cells were then treated for 24 h with 10D7 (5 μg/ml) or isotype control IgG (5 μg/ml) before addition of gemcitabine (0.02 to 500 nM) and incubation for another 72 h. In assays assessing the effect of cytotoxin-conjugated antibodies, cells were treated for 12 h with 10D7-MMAE, IgG-MMAE, 10D7 or IgG (0.0625 to 1.0 μg/ml) then washed before the media was replaced with standard growth medium for 72 h. Relative cell viability was then measured by adding CellTiter AQueous One Solution Reagent to each well and measuring absorbance at 490 nm as described above. In co-culture assays, mKO2 expressing TKCC05 PDAC cells (2,000 cells/well) were co-cultured with GFP expressing hPSCs (2,000

cells/well) for 24 h in a 1:1 mixture of TKCC05 and hPSC growth medium before treatments with 10D7-MMAE, IgG-MMAE, 10D7 or IgG (0.0625 to 1.0 µg/ml), IgG or 10D7 (1 µg/ml) or PBS as above. Cells were imaged by wide-field fluorescence microscopy and the total area of confluence for each cell type was quantified by image analysis using ImageJ software.

Radio-labelling of 10D7 with Zr^{89}

10D7 and control IgG1κ were labelled with the positron-emitting radionuclide ^{89}Zr as described [44]. Yield and purity of the labelled antibodies were determined by radio-thin layer chromatography and - high performance liquid chromatography (Agilent, Mulgrave, Australia). To assess the impact of radiolabelling on 10D7 binding, the immune-reactive fraction (IRF) of 10D7-^{89}Zr was determined by Lindmo assay as previously described [45]. Briefly, serially diluted TKCC05 cells (5×10^6 – 0.156×10^6 cells) were incubated with various amounts of 10D7-^{89}Zr alone or in the presence of a saturating amount of unlabelled 10D7 antibody (700 nM). After incubation (3 h at 4°C) cells were centrifuged and the radioactivity of the cell pellet and supernatant was determined using a 2480 Wizard Automatic Gamma Counter (Perkin Elmer, Glen Waverley, Australia) and IRF was calculated as previously described [45,46].

Bioluminescence imaging

Tumor development was monitored by *in vivo* bioluminescence imaging using an IVIS Spectrum (Perkin Elmer). Mice were injected intraperitoneally with D-luciferin diluted in PBS (15 mg/ml stock) at 150 mg/kg, anaesthetised and typically imaged 15-20 minutes after injection with D-luciferin. Bioluminescence was analysed using Living Image software (Perkin Elmer).

Figure S1.

CDCP1 expression in PDAC tumors. **A and B,** Kaplan-Meier analysis showing association between CDCP1 mRNA expression levels and PDAC patient survival in TCGA (n=170, A) and ICGC (n=267, B) datasets. Patients in each dataset were segregated into low and high CDCP1 expressing groups based on median, first quartile or third quartile of CDCP1 mRNA expression level. **C.** Association analysis between CDCP1 expression level and tumor size or tumor stage performed on the ICGC cohort. Statistical analysis was performed using ANOVA test. **C,** Kaplan-Meier analysis showing association between CDCP1 protein expression levels and PDAC patient survival in the ICGC-PACA-AU (n=222) cohort. For this analysis patients with CDCP1 expression at or below the quartile score were segregated into "low" and those with expression above the median or quartile score were segregated into the "high" CDCP1 expressing group. Statistical differences between Kaplan-Meier curves were determined by Mantel-Cox test.

Figure S2.

A, Densitometry analysis of the relative proportion of CDCP1-FL (130 kDa) and CDCP1-CTF (70kDa) based on western blot analyses performed on PDAC cells using anti-CDCP1 antibody 4115 and an anti-GAPDH antibody. **B,** Anti-CDCP1 western blot analysis with the indicated antibodies of proteins immunoprecipitated with antibody 4115 or isotype matched control antibodies from TKCC02 and TKCC05 cell lysates. **C,** Anti-CDCP1 western blot analysis with the indicated antibodies of proteins from cell lysates (C) or conditioned medium (CM, top panel) or from cell fractionation into cell surface (CS) or intracellular (Int) fractions prepared using by cell surface biotinylation protocol (bottom panel) of TKCC05 cells stably expressing non-targeting ShRNA (ShControl) or CDCP1-targeting ShRNA (ShCDCP1#1). **D,** Western blot analysis using anti-CDCP1 antibodies 4115 and 2666, and an anti-GAPDH antibody, of PANC-1, TKCC02, TKCC05 and TKCC10 cell lysates under reducing (left) and non-reducing (right) conditions.

Figure S3.

Hematoxylin and eosin staining (left) and CDCP1 immunohistochemistry (right, antibody 4115) of xenografts of PANC-1, TKCC02, TKCC05 and TKCC10 PDAC cells grown subcutaneously in mice.

Fig S4.

Function blocking antibody 10D7 induces rapid phosphorylation and degradation of differentially cleaved CDCP1 in PDAC cells. **A,** Western blot analysis of lysates of PANC-1 (*left*) and TKCC02 (*right*) cells treated for up to 300 min with anti-CDCP1 antibody 10D7 or isotype matched IgG. Lysates were probed for p-CDCP-Y734, CDCP1 (antibody 4115), p-Src-Y417, Src and β-actin. **B,** Western blot analysis of lysates of PANC-1 (*left*) and TKCC02 (*right*) cells treated for longer periods with anti-CDCP1 antibody 10D7 or isotype matched IgG. Lysates were probed for CDCP1 (antibody 4115) and β-actin.

Figure S5.

Total and cell surface expression of CDCP1 in PDAC cells. **A,** Western blot analysis of lysates of PANC-1, TKCC02, TKCC05 and TKCC10 PDAC cells stably expressing CDCP1-shRNA (shCDCP1 #1 and #2) or scramble shRNA (shControl). Lysates were probed for CDCP1 (antibody 4115) and GAPDH. Lysates from PANC1 and TKCC05 cells were also probed for CDCP1 using antibody 2666. **B,** Quantification of CDCP1 receptor number on the cell surface by flow cytometry analysis of PANC-1, TKCC02, TKCC05 and TKCC10 PDAC cells stably expressing ShCDCP1 #1 or ShControl. Number of receptor (median) for each cell lines are indicated on the right for ShControl cells and on the left for ShCDCP1 #1 cells.

Figure S6.

CDCP1 expression in PDAC mouse xenografts. **A,** Immunohistochemical staining for CDCP1 (antibody 4115) of representative PANC-1 xenograft tumors from mice treated with PBS, IgG or 10D7. **B,** Densitometry analysis of relative level of CDCP1 expression performed on western blot analysis of lysates of xenograft tumors from experiments presented in Figure 6A and B using antibody 4115. **C and D,** Western blot analysis of lysates from subcutaneous xenografts from PANC-1 (B) and TKCC05 (C) cells stably expressing control ShRNA (ShControl) or CDCP1 ShRNA (ShCDCP1). Lysates were probed for CDCP1 (antibody 4115) and GAPDH. **E,** Statistical significance of the Kaplan-Meier survival analysis shown in Figure 6E was performed using Log-rank Gehran-Breslow Wilcoxon Chi^2 test.

Supplementary Table S1. Clinical characteristics of patient cohorts

	**Transcriptomic**		**IHC**
**Clinical/Pathological Features**	**TCGA-PAAD (*n* = 170)**	**ICGC-PACA-AU (n=267)**	**ICGC-PACA-AU (n=222)**
**Age at diagnosis** (avg. (min, max)	62.77 (47.38, 86.01)	66.89 (34, 90)	66.36 (34, 88)
**Sex** (male proportion (F, M))	44.12 (95, 75)	53.18 (125, 142)	50.9 (109, 113)
**Ethnicity**			
White/Caucasian	150	237	200
Asian	10	17	17
Black/African	6	3	3
Other	4	10	2
**Stage**			
IA	0	4	5
IB	1	11	3
IIA	120	44	43
IIB	49	185	143
III	0	1	1
IV	0	12	11
Unknown	0	10	16
**Pathology TNM**			
T1	0	5	5
T2	15	27	6
T3	155	223	191
T4	0	1	1
Unknown	0	11	19
N (N1 proportion (N0, N1))	71.18 (49, 121)	76.86 (59,196)	74.75 (51, 151)
M (M0 proportion (M0, MX))	70.59 (120, 50)	M0: 7, M1:12, MX: 248	M0: 8, M1:11, MX: 203

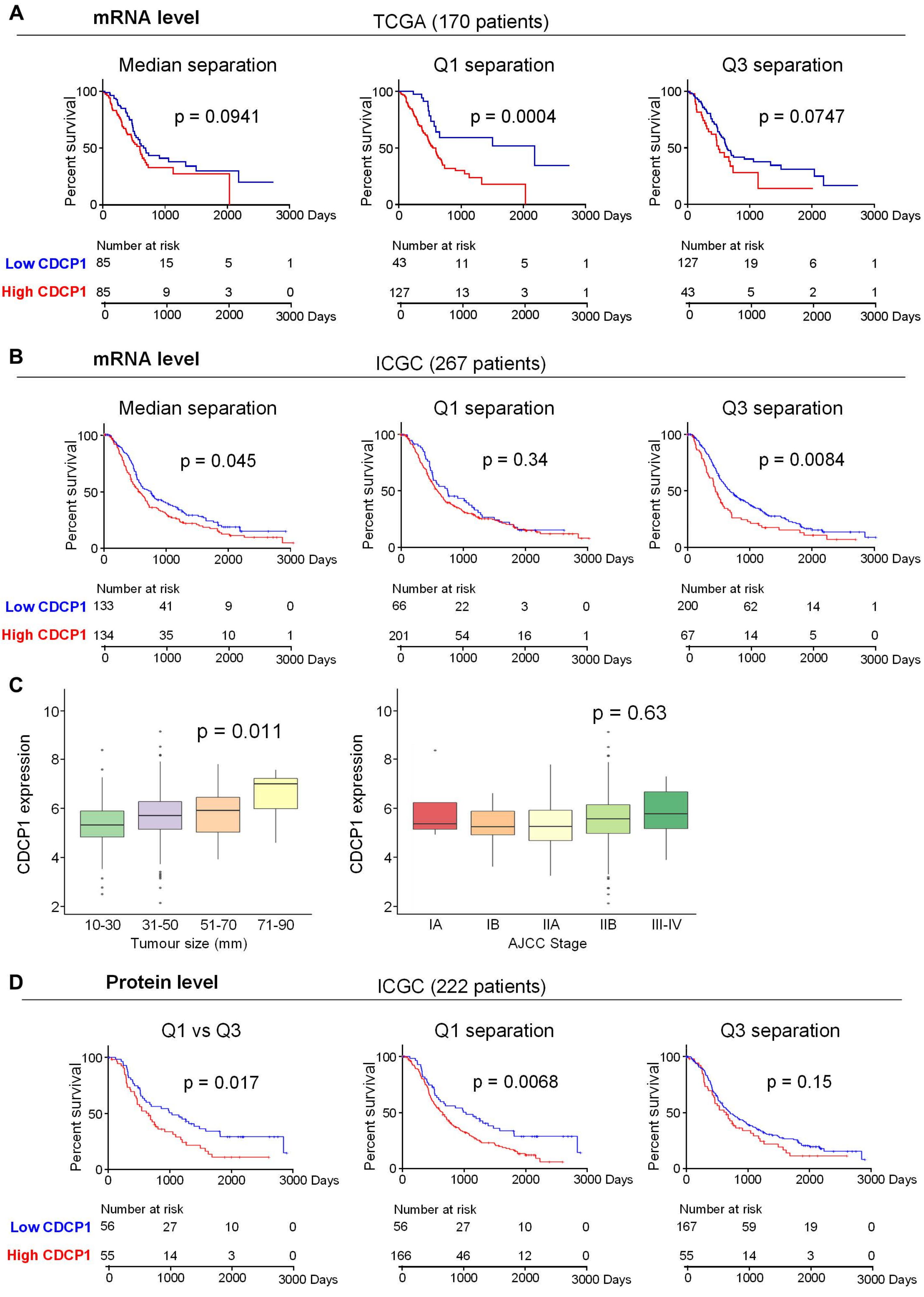
A
mRNA level
TCGA (170 patients)
Median separation
p = 0.0941
Q1 separation
p = 0.0004
Q3 separation
p = 0.0747
Percent survival
Days
Number at risk
Low CDCP1 85 15 5 1
High CDCP1 85 9 3 0
Low CDCP1 43 11 5 1
High CDCP1 127 13 3 1
Low CDCP1 127 19 6 1
High CDCP1 43 5 2 1
B
mRNA level
ICGC (267 patients)
Median separation
p = 0.045
Q1 separation
p = 0.34
Q3 separation
p = 0.0084
Percent survival
Days
Number at risk
Low CDCP1 133 41 9 0
High CDCP1 134 35 10 1
Low CDCP1 66 22 3 0
High CDCP1 201 54 16 1
Low CDCP1 200 62 14 1
High CDCP1 67 14 5 0
C
CDCP1 expression
p = 0.011
10-30
31-50
51-70
71-90
Tumour size (mm)
p = 0.63
IA
IB
IIA
IIB
III-IV
AJCC Stage
D
Protein level
ICGC (222 patients)
Q1 vs Q3
p = 0.017
Q1 separation
p = 0.0068
Q3 separation
p = 0.15
Percent survival
Days
Number at risk
Low CDCP1 56 27 10 0
High CDCP1 55 14 3 0
Low CDCP1 56 27 10 0
High CDCP1 166 46 12 0
Low CDCP1 167 59 19 0
High CDCP1 55 14 3 0

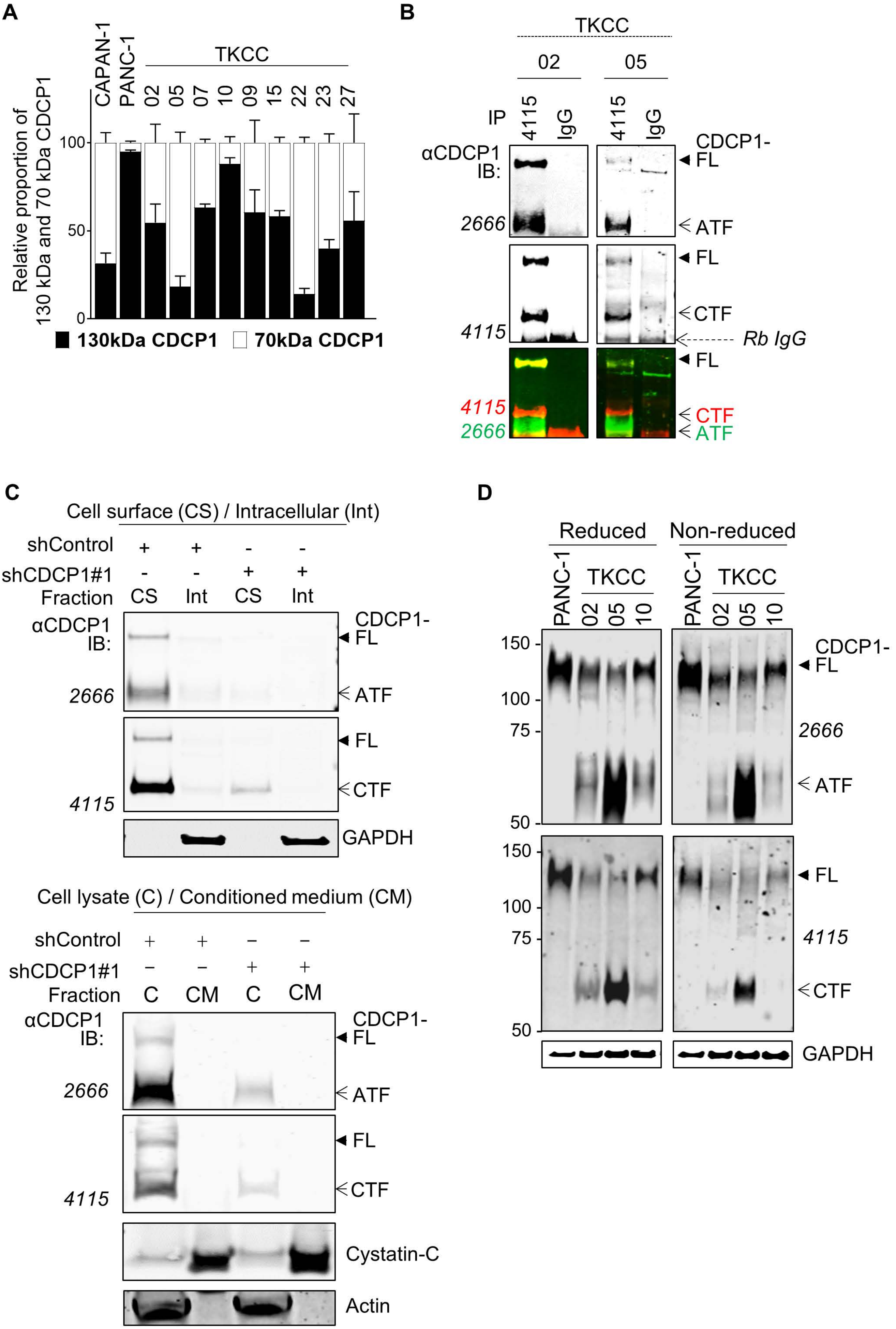

A
B
C
D
CAPAN-1
PANC-1
TKCC
02 05 07 10 09 15 22 23 27
Relative proportion of 130 kDa and 70 kDa CDCP1
130kDa CDCP1
70kDa CDCP1
IP
4115
IgG
αCDCP1 IB:
2666
4115
CDCP1-FL
ATF
CTF
Rb IgG
Cell surface (CS) / Intracellular (Int)
shControl
shCDCP1#1
Fraction
CS
Int
GAPDH
Cell lysate (C) / Conditioned medium (CM)
C
CM
Cystatin-C
Actin
Reduced
Non-reduced
150
100
75
50

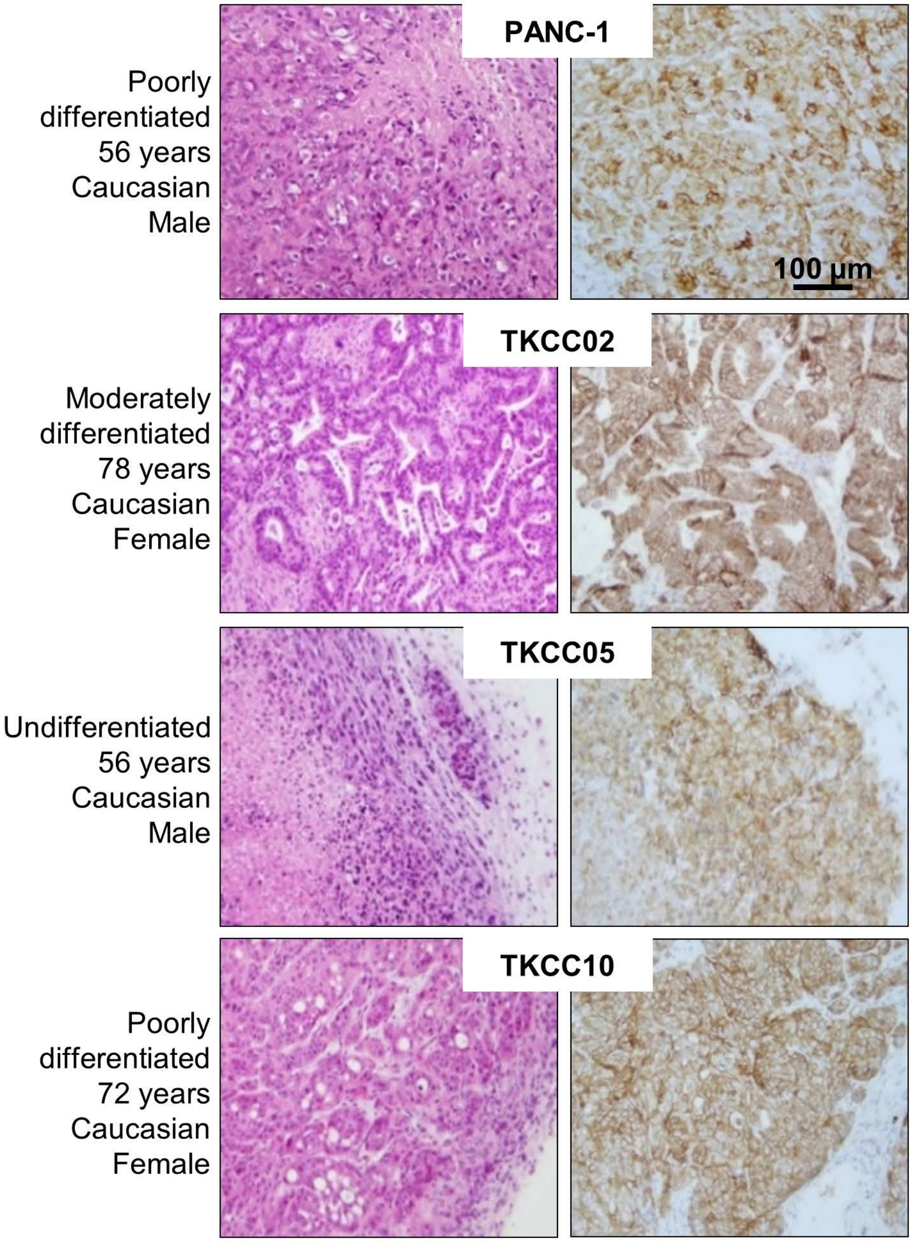
PANC-1
Poorly differentiated
56 years
Caucasian
Male
100 µm
TKCC02
Moderately differentiated
78 years
Caucasian
Female
TKCC05
Undifferentiated
56 years
Caucasian
Male
TKCC10
Poorly differentiated
72 years
Caucasian
Female

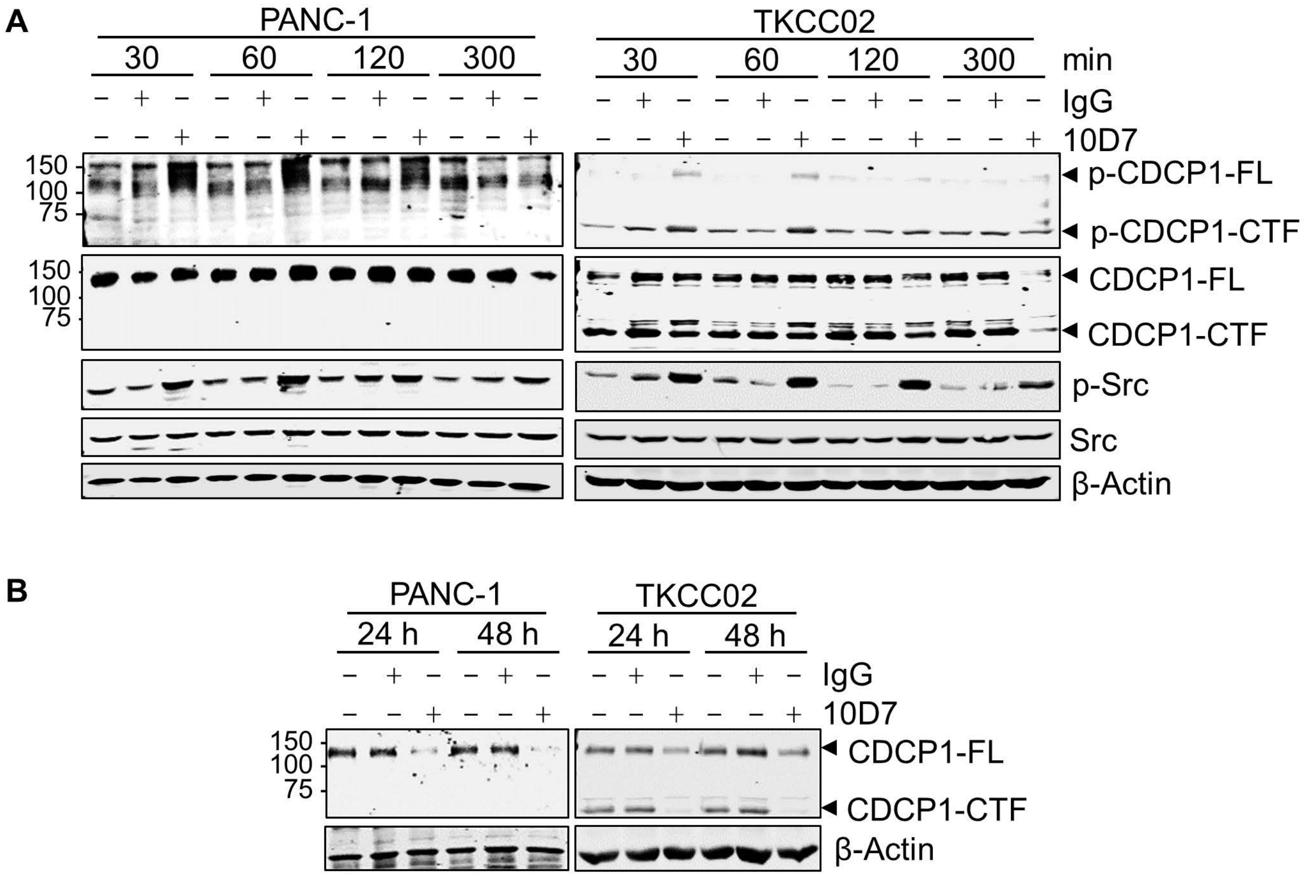
A
PANC-1
TKCC02
30
60
120
300
min
IgG
10D7
150
100
75
p-CDCP1-FL
p-CDCP1-CTF
CDCP1-FL
CDCP1-CTF
p-Src
Src
β-Actin
B
PANC-1
TKCC02
24 h
48 h
IgG
10D7
150
100
75
CDCP1-FL
CDCP1-CTF
β-Actin

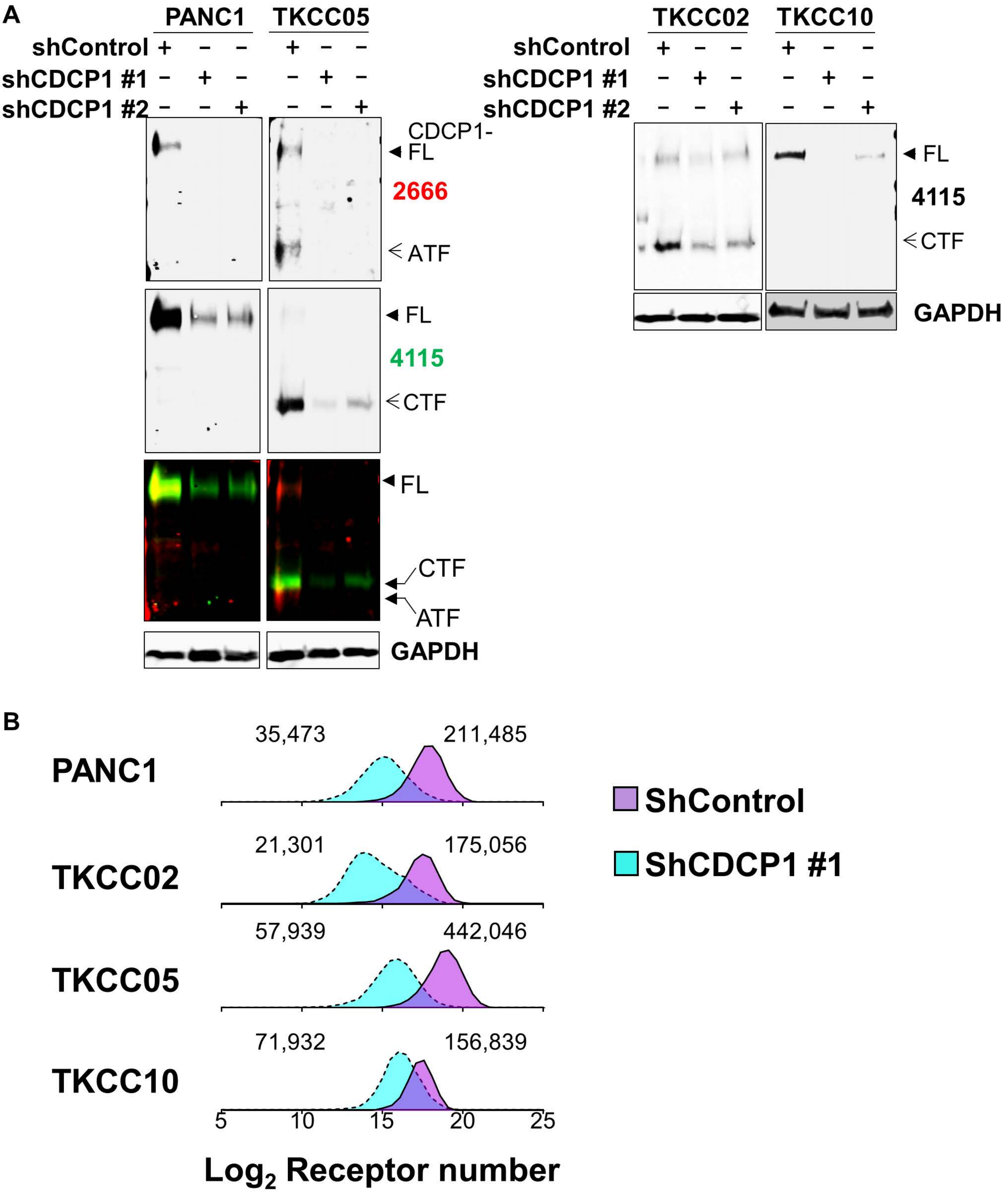
A
PANC1
TKCC05
TKCC02
TKCC10
shControl
shCDCP1 #1
shCDCP1 #2
CDCP1-
FL
2666
ATF
4115
CTF
GAPDH
B
PANC1
TKCC02
TKCC05
TKCC10
35,473
211,485
21,301
175,056
57,939
442,046
71,932
156,839
5
10
15
20
25
Log2 Receptor number
ShControl
ShCDCP1 #1

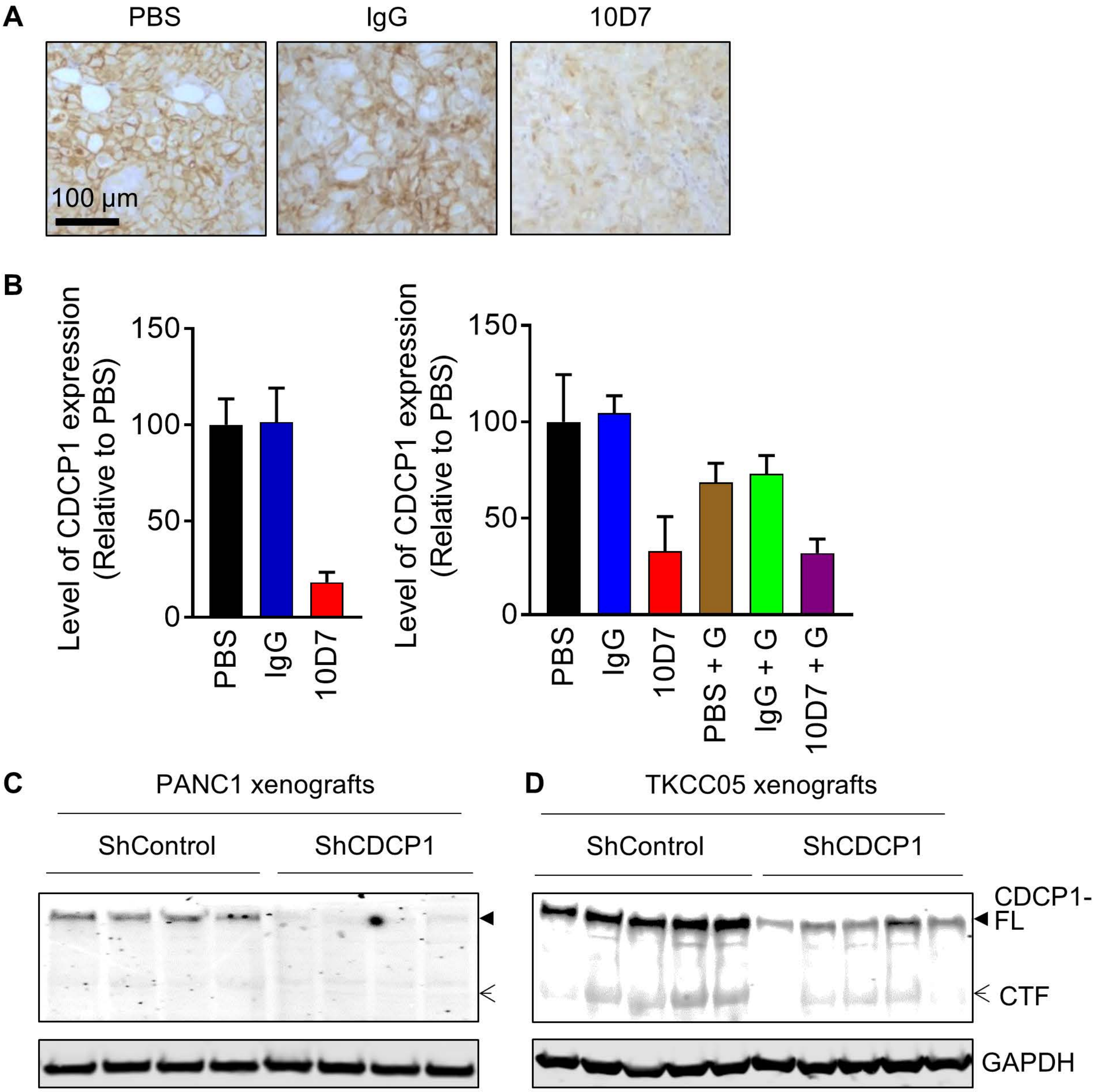

E

	PBS	10D7	Gem	IgG-MMAE	10D7-MMAE
PBS		0.0460	0.0023	0.8704	0.0014
10D7	0.0460		0.0303	0.0567	0.0014
Gem	0.0023	0.0303		0.0021	0.0014
IgG-MMAE	0.8704	0.0567	0.0021		0.0013
10D7-MMAE	0.0014	0.0014	0.0014	0.0013	
